# Congener-specific partition properties of chlorinated paraffins evaluated with COSMO*therm* and gas chromatographic retention indices

**DOI:** 10.1038/s41598-021-84040-z

**Published:** 2021-02-24

**Authors:** Jort Hammer, Hidenori Matsukami, Satoshi Endo

**Affiliations:** Center for Health and Environmental Risk Research, National Institute for Environmental Studies (NIES), Onogawa 16-2, Tsukuba, Ibaraki 305-8506 Japan

**Keywords:** Structure prediction, Computational chemistry, Analytical chemistry, Environmental chemistry

## Abstract

Chlorinated Paraffins (CPs) are high volume production chemicals and have been found in various organisms including humans and in environmental samples from remote regions. It is thus of great importance to understand the physical–chemical properties of CPs. In this study, gas chromatographic (GC) retention indexes (RIs) of 25 CP congeners were measured on various polar and nonpolar columns to investigate the relationships between the molecular structure and the partition properties. Retention measurements show that analytical standards of individual CPs often contain several stereoisomers. RI values show that chlorination pattern have a large influence on the polarity of CPs. Single Cl substitutions (–CHCl–, –CH_2_Cl) generally increase polarity of CPs. However, many consecutive –CHCl– units (e.g., 1,2,3,4,5,6-C_11_Cl_6_) increase polarity less than expected from the total number of –CHCl– units. Polyparameter linear free energy relationship descriptors show that polarity difference between CP congeners can be explained by the H-bond donating properties of CPs. RI values of CP congeners were predicted using the quantum chemically based prediction tool COSMO*thermX*. Predicted RI values correlate well with the experimental data (R^2^, 0.975–0.995), indicating that COSMO*thermX* can be used to accurately predict the retention of CP congeners on GC columns.

## Introduction

Chlorinated Paraffins (CPs) are a group of substances that are applied in various products as plasticizers, coolants and flame retardants because of their chemical and thermal stability^1^. CPs are high-volume production chemicals (> 1 million metric tonnes yr^−1^) and are regularly released into the environment during production, transportation, and recycling processes and through leaching and volatilization from landfills^2–4^. Short-chain chlorinated paraffins (SCCPs; C_10_-C_13_) are found to be persistent, bioaccumulative and toxic (PBT) to aquatic organisms. In 2017, SCCPs were classified as persistent organic pollutants (POPs) under the Stockholm Convention and subsequently the production of SCCPs has stopped in the US, Japan, Canada and Europe, and will soon be restricted in China^5,6^. Since the PBT properties of medium-chain (MCCPs: C_14_-C_17_) and long-chain (LCCPs; C_18_ and longer) chlorinated paraffins are less studied and a matter of debate, they are currently still being produced and used as alternatives for SCCPs^7^. Therefore, the overall world-wide production of CPs still upholds its increasing trend from the 1950s, albeit with a recent shift from SCCPs towards MCCPs and LCCPs.

CP molecules are usually produced by free-radical chlorination of *n*-alkanes. This chlorination reaction shows low positional selectivity and produces many congeners and isomers and does not discriminate between stereoisomers^8^. CP mixtures can therefore comprise thousands of congeners with differing chain lengths and chlorination patterns. The large variability in molecular structure suggests that intermolecular interaction properties also vary substantially. Intermolecular interactions determine the partitioning behavior of CPs and need to be understood to describe the environmental fate, bioaccumulation, and toxicity of CPs. The broad bands of CP signals observed in chromatographic analysis do suggest that congeners have a range of partition properties^9^.

The objective of this work is to describe the relationship between structure and molecular interaction properties of CPs through experimental and quantum chemically based approaches. A total of 25 available analytical standards of CPs with chain length C_10_ or longer were selected for the current study. Gas chromatography (GC) was used to experimentally investigate the molecular interaction properties, as the retention time of the analyte on a GC column is directly related to the molecular interactions between the column coating and the analyte molecule. Different GC column coatings were selected with a range of polarity to elucidate the polar interaction properties of CPs. The physico-chemical properties of CP congeners were evaluated by deriving poly-parameter linear free energy relationship (ppLFER) descriptors from the measured data. In the following discussions, we initially define “polarity” and “polar interactions” as those that increase GC retention on polar columns as compared to nonpolar columns. We then investigate through the ppLFER the types of polar interactions that are responsible for the increased retention. The quantum-chemically based tool COSMO*thermX* (COSMO*logic* GmbH & Co. KG) was used to predict partition coefficients and retention times of CPs on all columns. COSMO*thermX* has previously been used to predict partition coefficients such as octanol–water partition coefficients for CPs^10,11^. Because COSMO*thermX* requires only the molecular structure as input parameter, it could be a useful tool to predict partition properties of CP congeners with diverse structures.

## Methods

### Chemicals

Analytical standards of 2,5,6,9-C_10_Cl_4_, 1,2,5,6,9,10-C_10_Cl_6_ and 2,3,4,5,6,7,8,9-C_10_Cl_8_ were provided by Dr. Ehrenstorfer GmbH (Augsburg, Germany). Standards of 1,1,1,3-C_10_Cl_4_, 1,1,1,3-C_11_Cl_4,_ 1,1,1,3-C_12_Cl_4,_ 1,1,1,3-C_13_Cl_4,_ 1,1,1,3-C_14_Cl_4,_ 1,1,1,3,9,10-C_10_Cl_6_, 1,1,1,3,10,11-C_11_Cl_6_, 1,1,1,3,11,12-C_12_Cl_6_, 1,1,1,3,12,13-C_13_Cl_6_, 1,1,1,3,8,10,10,10-C_10_Cl_8_, 1,1,1,3,9,11,11,11-C_11_Cl_8_, 1,1,1,3,10,12,12,12-C_12_Cl_8_, 1,1,1,3,11,13,13,13-C_13_Cl_8_, 1,1,1,3,12,14,14,14-C_14_Cl_8_, 1,2,9,10-C_10_Cl_4_, 1,2,10,11-C_11_Cl_4_, 1,2,13,14-C_14_Cl_4_, 1,2,3,4,5,6-C_11_Cl_6_, 4,5,7,8-C_11_Cl_4_, 2,3,4,5-C_10_Cl_4_ and 2,3,4,5-C_12_Cl_4_ were obtained from Chiron AS (Trondheim, Norway). 1,5,5,6,6,10-C_10_Cl_6_, which was commercially available from Cambridge Isotope Laboratories Inc. (Tewksbury, MA, USA), was donated by Otsuka Pharmaceutical Co., Ltd. (Tokyo, Japan). C_16_, C_18,_ C_20_-*n*-alcohols, a mixture of C_7–40_-*n*-alkanes and a mixture of C_4_, C_6_, C_8_, C_10_, C_12_, C_14_, C_16_, C_18_, C_20_, C_22_, C_24_-methyl esters (FAMEs) were obtained from Sigma-Aldrich Japan (Tokyo, Japan). C_8_, C_10,_ C_12_-*n*-alcohols were obtained from Tokyo Chemical Industry (Tokyo, Japan). A mixture of polycyclic aromatic hydrocarbons (PAHs) containing naphthalene, acenaphthylene, acenaphthene, fluorene, phenanthrene, anthracene, fluoranthene, pyrene, benz[*a*]anthracene, chrysene, benzo[*b*]fluoranthene, benzo[*k*]fluoranthene, benzo[*a*]pyrene, dibenzo[*a*,*h*]anthracene, indeno[1,2,3-*cd*]pyrene and benzo[*ghi*]perylene was obtained from Sigma-Aldrich Japan (Tokyo, Japan). Specifics on purities and concentrations of the CP analytical standards can be found in Supplementary Table S1.

We note that studies have shown that the current analytical standards often include chlorine substitution patterns that are not commonly present in technical mixtures. For example, triple-chlorine substitution at the terminal carbon atom (e.g., 1,1,1,3,9,10,10,10-C_10_Cl_7_) is usually not found in technical mixtures and environmental samples^12^. A more distributed chlorination pattern (e.g., 2,5,6,9-C_10_Cl_4_, 2,3,4,5,6,7,8,9-C_10_Cl_8_) may have higher relevance, although the availability of such standards is limited. In this work, we considered all available analytical standards as probes, irrespective of their relevance, to keep the scope large in terms of structural variability.

### Columns

Six GC columns were used for the retention measurements in this study (Table 1). The GC columns were selected to cover a wide range of polarity based on polarity scales provided by manufacturers. The SPB-Octyl column is of nonpolar nature and the least polar column in this study. Its coating consists of poly(50% *n*-octyl/50% methylsiloxane) and exerts retention mainly via van der Waals interactions. The polar property of columns HP-5ms, InertCap-17ms and DB-17ms originates from the presence of phenyl groups in the dimethylsiloxane (HP-5ms and InertCap-17ms) or silarylene-siloxane polymer (DB-17ms) structure of the column coating. These columns contain 5% or 50% phenyl groups. Phenyl groups have *π* electrons that have weak hydrogen (H)-bond accepting properties. The DB-225ms column, with a coating of 50% cyanopropylphenyl/50% dimethylsiloxane-equivalent silarylene-siloxane copolymer, contains a polar nitrile group that acts as a H-bond acceptor. The polar property of the SolGel-WAX column originates from the ether oxygen atoms in poly(ethylene glycol), which has strong H-bond accepting properties. All columns had the dimension of 30 m × 0.25 mm × 0.25 μm.

**Table 1:** Polymer coating compositions of the GC columns and structures of the surrogate molecules used in COSMO*thermX*. The circled parts (red) on the molecular fragments refer to the groups that were disregarded using the weight string function in COSMO*thermX*.

GC system					
Column	Coating composition according to manufacturer	Manufacturer	GC oven temperature program	GC system/detection	Fragments representing the polymer phase in COSMO*thermX*
SPB-Octyl	Poly(50% n-octy/50% dimethylsiloxane)	Supelco	70 °C (1 min) 10 °C/min 280 °C (10 min)	Agilent 7890 GC/Agilent 6530 QTOF-MS	
HP-5ms	Poly(5% diphenyl/ 95% dimethylsiloxane)	Agilent Technologies	70 °C (0.1 min) 10 °C/min 280 °C (10 min)	Agilent 7890A GC/Agilent 7000A Triple Quad GC/MS	
DB-17ms	Poly(50% phenyl/50% dimethylsiloxane)^1^	Agilent Technologies	60 °C (1 min) 10 °C/min 300 °C (10 min)	Agilent 7890 GC/Agilent 6530 QTOF-MS	
InertCap-17ms	Poly(50% diphenyl/50% dimethylsiloxane)	GL Sciences	70 °C (1 min) 20 °C/min 300 °C (10 min)	Agilent 7890A GC/Agilent 7000A Triple Quad GC/MS	
DB-225ms	Poly(50% cyano-propylphenyl/ 50% dimethylsiloxane) ^1^	Agilent Technologies	70 °C (0.1 min) 10 °C/min 240 °C (15 min)	Agilent 7890 GC/Agilent 6530 QTOF-MS	
SolGel-WAX	Polyethylene glycol	SGE Analytical Science	70 °C (1 min) 10 °C/min 280 °C (5 min)	Agilent 7890 GC/Agilent 6530 QTOF-MS	

^1^ Silarylene-siloxane copolymer; ^2^ Instead of 5 and 95% mole fractions, 5.3 and 94.7% was used as the liquid phase composition for HP-5ms in COSMO*thermX* since the larger fragment contains not only diphenylsiloxane, but dimethylsiloxane as well.

### Retention measurements

A program with linear oven temperature increase was applied on all columns until the recommended maximum temperature was reached (240–300 °C). Helium was used as carrier gas and a flow rate of 1.2 mL/min was maintained throughout all measurements. Retention measurements for SPB-Octyl, DB-17ms, DB-225ms, and SolGel-WAX were performed using cool on-column injections on an Agilent 7890 GC, followed by atmospheric pressure chemical ionization (APCI) and mass selective detection (Agilent 6530 QTOF-MS) (See Supplementary Section S2 for the optimization of APCI-QTOF-MS parameters for CPs). An injection volume of 2 μL was used. The on-column injector temperature was kept at the initial oven temperature (60 or 70 °C) for 0.1 min and increased with 100 °C/min to the maximum oven temperature. The oven temperature was kept at 60 or 70 °C for 0.1 min and increased with 10 °C/min to the maximum temperature shown in Table 1. More details are stated in the Supplementary Section S2. Retention measurements of SPB-Octyl and SolGel-WAX were also performed on a different system (7890A/7000A triple quadrupole GC/MS, Agilent Technologies) and HP-5ms and InertCap-17ms only on this system because of its better availability in our laboratory. On the triple quadrupole GC/MS system, splitless injection at 250 °C and electron ionization (EI) were used. Peak patterns were similar on both systems, and the retention indices (RIs; see below for the definition) differed only by 7 on average and 20 in the worst case. In contrast to the EI-MS detector, the APCI-QTOF-MS method allows for a detection of pseudo-molecular ions and thus better identification of peaks that belong to the stated CP isomers. Therefore, if the measurements were done on both systems, data from APCI-QTOF-MS were considered for the latter discussions. Peak identifications for the EI-MS chromatograms of HP-5ms and InertCap-17ms were performed by using the APCI-TOF-MS chromatograms of SPB-Octyl and DB-17ms, respectively, as reference, because the peak patterns were highly similar (see the results section).

RIs of CPs, *n*-alcohols, *n*-alkylmethyl esters, PAHs and *n*-alkanes were obtained using the linear temperature-programmed retention index system (LTPRI). This system is used to establish retention indices for retention times measured under a program with linear temperature increase^13,14^:

1 $$ RI = \frac{{Rt_{i} - Rt_{x} }}{{Rt_{x + 1} - Rt_{x} }} \times 100 + RI_{x} $$

where *Rt*_i_ is the retention time of the analyte, *Rt*_x_ is the retention time of the *n*-alkane eluting directly before *Rt*_i_, *Rt*_x+1_ is the retention time of the *n*-alkane eluting directly after *Rt*_i_ and RI_x_ is the retention index of the *n*-alkane that corresponds to *Rt*_x_. The retention indices of *n*-alkanes are defined as its number of carbon atoms times a hundred.

### ppLFER descriptors

ppLFERs are useful in characterizing interaction properties that determine partitioning behavior of chemicals. ppLFERs are multiple linear regression models that use several solute descriptors as independent variables for the calculation of partition coefficients^15^. The most frequently used ppLFER for the gas-condensed phase partitioning, established by Abraham et al.^16^ has the general form:

2 $$ \log K = c + eE + sS + aA + bB + lL $$

where log *K* is the logarithmic partition coefficient. The uppercase letters on the right-hand side of the equation are the solute descriptors: *E*, excess molar refraction; *S*, dipolarity/polarizability parameter; *A*, H-bond donating property; *B*, H-bond accepting property and *L*, logarithmic hexadecane-air partition coefficient. The lowercase letters are the system parameters. Each term quantitatively describes the energetic contribution of a molecular interaction to log *K*. Since none of the columns from the current study has H-bond donating properties, the *bB* term can be ignored. Solute descriptors *S* and *A* are both responsible for the polar interactions of the chemical: *S* is related to the surface electrostatic property and is thought to represent polar interactions that result in part from the partial charge distribution over the molecular surface^17^. *A* reflects more specific interactions resulting from H-bond donating sites of the solute molecule. The *L* solute descriptor describes the non-specific van der Waals interactions and also includes the energy needed for cavity formation^15,18^. The *eE* term also describes the van der Waals interactions but usually has only minor contributions to log *K*. For more detailed explanations of the equation, we refer to Refs.^13–15^.

In this study, temperature-programmed RIs instead of log *K* are correlated with the ppLFER descriptors. Because temperature-programmed RI is related but not directly proportional to log *K*^19^, the use of ppLFER for the RI is an approximation. For a more accurate investigation, isothermal retention measurements would be better suited, although much more time-consuming than temperature-programmed measurements, as isothermal measurements must be performed at many temperatures to cover diverse CP structures. The purpose of using ppLFERs in the current work is to compare semi-quantitatively the polar interaction properties of CP congeners with varying structures and not to derive accurate solute descriptors that could be used for later predictions.

### Prediction of RI with COSMOthermX

COSMO*thermX* software is based on the COSMO-RS theory, which uses quantum mechanics and statistical thermodynamics calculations to determine the chemical potential of a solute in solution and can thereby predict partition coefficients. Gas-GC coating (i.e., air-polymer) partition coefficients were predicted following the method by Goss^20^. Molecular structures of CPs, reference compounds and polymer coatings were expressed with SMILES strings, which were then converted to SDF files. Quantum chemical calculations and conformer selection were performed using COSMO*confX* (version 4.3, COSMO*logic*) with TURBOMOL 7.3, which yield a complete set of relevant conformations with full geometry optimization in the gas phase and in the conductor reference state. The gas phase energy and COSMO files of the CPs and reference compounds were then used in the COSMO*thermX* software (version 19.04; parameterization: BP_TZVPD_FINE_19) to calculate air-polymer partition coefficients (*K*_air-polymer_). To represent the molecular structure of polymer coating, monomers or oligomers of the coating polymer structure provided by the manufacturer were used. For the quantum chemical calculations performed by COSMO*confX*, the end groups of these monomer or oligomer were end-capped with CH_3_ groups. The CH_3_ groups were later disregarded during COSMO*thermX* calculations by giving a weighting factor of 0, following the approach by Goss^20^. All these surrogate structures used for coating polymers are shown in Table 1. The polymer structure of the HP-5ms column consists of 5% diphenylsiloxane and 95% dimethylsiloxane and we represented this structure with a mixture of diphenylsiloxane and dimethylsiloxane in the respective mole fractions (see Table 1) in the COSMO*thermX* calculations. For the SolGel-WAX column, an end-capped trimer of ethylene glycol was used, as in Ref.^17^.

All calculations in COSMO*thermX* were performed with the combinatorial term switched off, as is recommended for polymer by the COSMO*thermX* user guide^20,21^. All conformers generated by COSMO*confX* of the target chemicals were used for the calculation of air-polymer partition coefficients. However, to reduce calculation times, only the top 5 low-energy conformers returned by COSMO*confX* (_c0 to _c4 suffixes) were selected to represent the polymer phases. For some CPs, COSMO*confX* returned conformers with *R* or *S* configurations that were inconsistent with the input structure. This problem did not occur when we turned off RDKit and only used Balloon for the generation initial conformers on the Windows version of COSMO*confX*.

For each chemical and coating phase, *K*_air-polymer_ was predicted at 5 temperature steps between 373.15 and 573.15 K. Then, linear regression between log *K*_air-polymer_ and 1/*T* was established, and a hypothetical eluting temperature was interpolated at a column-specific, characteristic *K*_air-polymer_ value that is derived from experimental data. This eluting temperature was considered analogous to the retention time and used to derive RI, following Eq. (2). A more detailed explanation about how RI values were predicted from COSMO*thermX* calculations is presented in the Supplementary Section S1.

Because the stereometric structure of the isomers present in the CP standards is unknown, partition coefficients were calculated for all possible diastereomers using COSMO*thermX*. A pair of enantiomers was represented by a single structure in the COSMO*thermX* calculation, because partition coefficients of enantiomers are the same in isotropic phases. The predictability of the COSMO*thermX* program was tested by comparing the mean of predicted RIs for all possible diastereomers and the weighted mean of the measured RI values of the CP standards from the GC system. RI values of PAHs were calculated but not used in testing the predictability of COSMO*thermX*, as their predicted RI values were systematically deviated from the measured values (see Supplementary Table S2).

## Results and discussion

### Determination of GC retention times and RI

**Figure 1:**
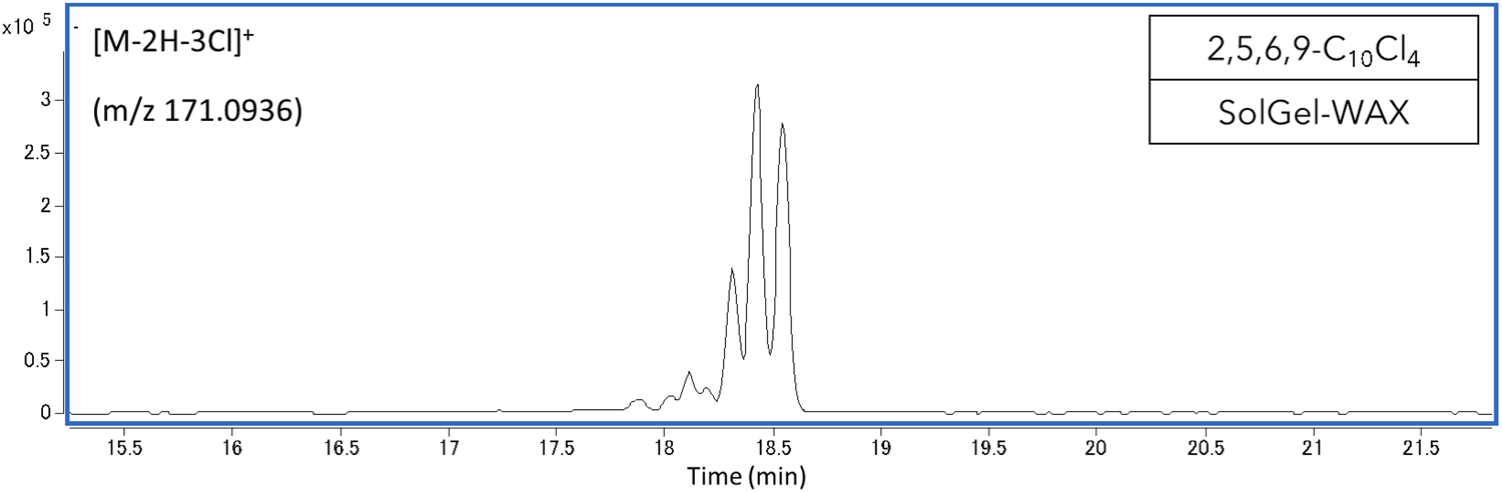
The Chromatogram of 2,3,6,9-C_10_Cl_4_ measured on the SolGel-WAX column. The manufacturer-provided certificate of analysis of this analytical standard stated the presence of three diastereomers.

Retention measurements showed the presence of multiple peaks in most of the CP analytical standards. Generally, CP congeners with a high number of possible diastereomers given their molecular structure showed multiple peaks with a substantial peak area of the same (pseudo)molecular ion. For example, on the SPB-Octyl column, 10 peaks within a minute of retention time were found for 1,2,3,4,5,6-C_11_Cl_6_, which has 10 possible diastereomers (a pair of enantiomers are considered one structure). In contrast, 1,1,1,3,9,10-C_10_Cl_6_ (2 possible diastereomers) only showed one peak (Supplementary Fig. S1A,B). For one standard, 2,5,6,9-C_10_Cl_4_ (6 possible diastereomers), the manufacturer-provided certificate of analysis stated the presence of three diastereomers without details on the exact stereometric structure (e.g., *S* and *R* notation). While we indeed observed three peaks on the nonpolar SPB-Octyl column, 7 peaks were found on the most polar SolGel-WAX column (Fig. 1), showing increased separation through polar interactions. As exceptional cases, 1,2,5,6,9,10-C_10_Cl_6_ (6 possible diastereomers) only showed one peak on all columns (Supplementary Fig. S1C) and 2,3,4,5,6,7,8,9-C_10_Cl_8_ (72 possible diastereomers) showed 3 peaks on the SPB-Octyl column, and only 1 peak on the SolGel-WAX (Supplementary Fig. S1D). These standards likely contain a limited number of diastereomers. Some CP standards with only few or no possible diastereomers resulted in a higher number of peaks. For example, 1,1,1,3-C_10_Cl_4_ (no diastereomer) showed 5 peaks over 3 min of retention time on the SPB-Octyl column (Supplementary Fig. S1E) and 4 peaks over 5 min of retention time on the SolGel-WAX column. Most of the peaks in these chromatograms were small and are likely constitutional isomers (i.e., impurities).

We note that no retention times of 1,1,1,3,11,12-C_12_Cl_6_, 1,1,1,3,9,10,10,10-C_10_Cl_8_ and 1,5,5,6,6,10-C_10_Cl_6_ could be determined on the SolGel-WAX column, as their peaks were broad and the response was low (Supplementary Fig. S1F). This peak broadening is probably because of thermal degradation^22^, as a high temperature (280 °C) was needed to elute these congeners. Indeed, 1,5,5,6,6,10-C_10_Cl_6_ and 1,1,1,3,9,10,10,10-C_10_Cl_8_ were detected on the other highly polar column DB-225ms, for which a lower temperature (240 °C) for elution was applied.

As a representative RI value for a CP congener with multiple peaks, the mean of the RI values weighted by the peak areas was calculated and used in the following discussions. While we are aware that peak areas do not always reflect the relative abundance of CP isomers present^23^, this approach deemed better than simply calculating the mean of RIs for all peaks without weighting, particularly in cases where one or a few major peaks appear with many small peaks.

### Comparison of RIs on polar and nonpolar columns

**Figure 2:**
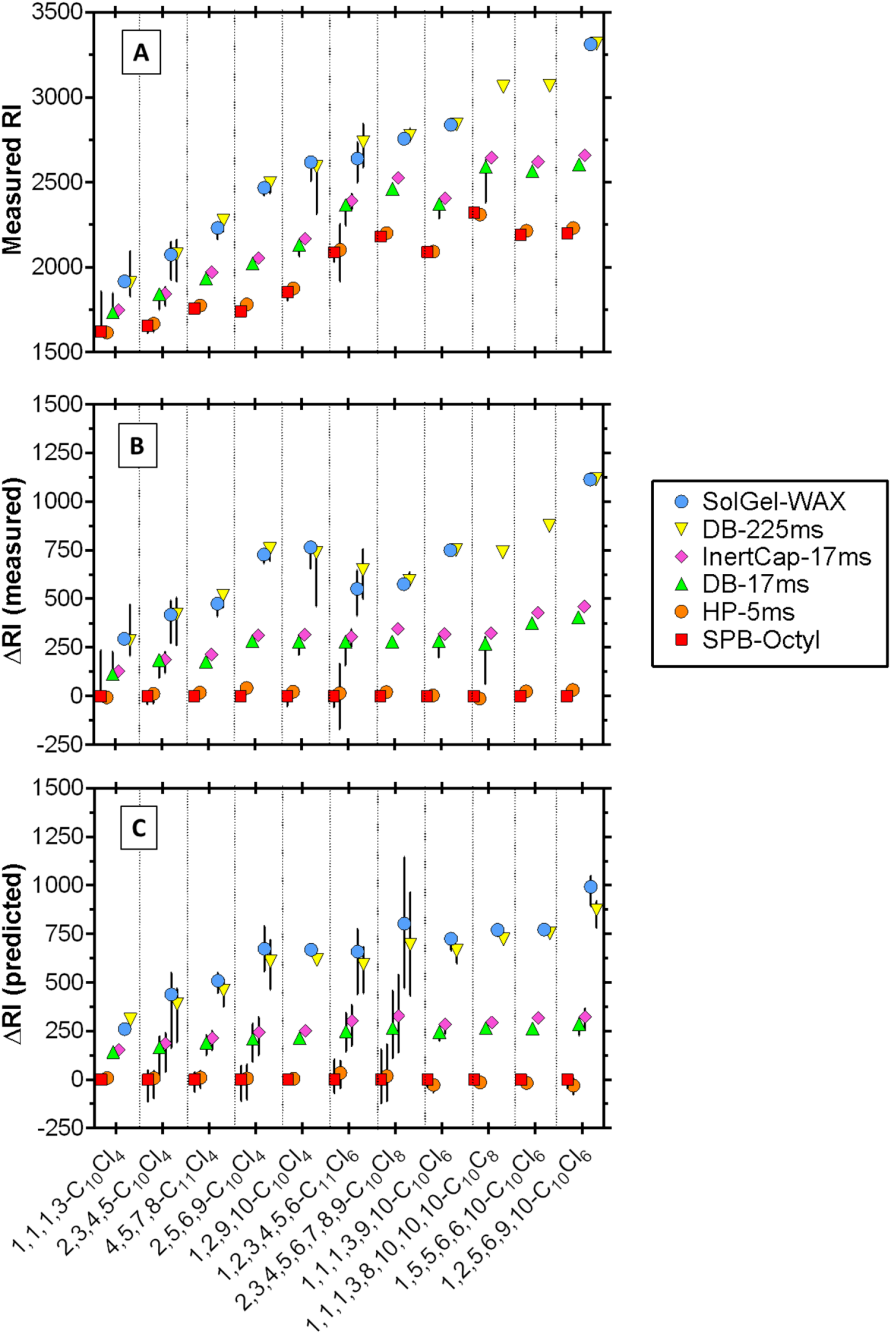
The measured RI on GC columns (**A**), the measured RI subtracted by the measured RI on the SPB-Octyl column (ΔRI) (**B**), and RI values predicted by COSMO*thermX* subtracted by predicted RI values of the SPB-Octyl column (**C**) for a selection of CP standards. The compounds are ordered according to the ΔRI for DB-225ms (polar column with data available for most CPs). The vertical error bars in panels A and B show the range of measured RIs for multiple peaks, while the vertical error bars in panel C show the range of predicted RI values for CPs with multiple diastereomers. Corrections were applied to predicted RIs (see text).

Since polar compounds are retained more by polar coatings, comparing RI values between columns of different polarity allows for the characterization of the polar interaction properties of CP molecules and substructures. RIs of CPs on polar columns were always higher than those on the nonpolar SPB-Octyl column, showing the significance of polar interaction properties for all CP standards (Fig. 2). The range of RIs (or separation of diastereomer/constitutional isomer peaks) for each CP standard was usually greater on polar columns, meaning that the isomers are better separated with polar retention mechanisms instead of van der Waals interactions only.

A series of CP congeners with -CH_2_- increments show that RI on all columns increased with 102 to 119 per addition of -CH_2_- to the alkyl chain. These values are similar to the RI values of *n*-alcohols (102–107 per methylene) and *n*-alkylmethyl esters (100–105 per methylene, see Supplementary Fig. S2).

Chlorine substitution on the alkyl chain generally increased RI to an extent depending on the column polarity and the position of Cl. For example, the RI of 1,2,9,10-C_10_Cl_4_ on the SPB-Octyl column is greater by 229 than that of a constitutional isomer 1,1,1,3-C_10_Cl_4_. The retention on the SPB-Octyl column is driven by van der Waals interactions, which are correlated to the molecular surface area of the molecule^24^. The four Cl atoms of 1,2,9,10-C_10_Cl_4_ are distributed over the alkyl chain and increase the molecular surface area more than the four Cl atoms of 1,1,1,3-C_10_Cl_4_ that are shifted on one side.

Figure 2B shows ΔRI, defined as the RI of a column subtracted by the RI of SPB-Octyl to clarify the contributions of polar interactions to the retention. Larger ΔRI values are observed with increasing column polarity, while the trends of ΔRI over different congeners are similar for all columns. Generally, a single Cl substitution on –CH_2_– to –CHCl– increases the polarity of CPs. However, actual contributions appear to depend strongly on the neighboring structure of the –CHCl– group. A vicinal substitution pattern (–CHCl–CHCl–) does not increase the polarity so much as an isolated –CHCl–. This is clearly shown with 2,3,4,5,6,7,8,9-C_10_Cl_8_, which shows only an intermediate ΔRI although having the highest number of –CHCl– units. In a vicinal substitution pattern, the proximity of –CHCl– groups might interfere with, and lower, the polarity and/or H-bond properties of a neighboring –CHCl– group. In contrast, a single Cl substitution on a terminal carbon (–CH_3_) is less influenced by Cl on the neighboring carbon. Comparison of the 5 tetrachloro (Cl_4_) congeners is illustrative for these trends: 2,5,6,9-C_10_Cl_4_ (2 isolated Cl and a pair of vicinal Cl) and 1,2,9,10-C_10_Cl_4_ (2 pairs of vicinal Cl at the ends) show the highest ΔRI, followed by 4,5,7,8- C_11_Cl_4_ (2 pairs of vicinal Cl) and 2,3,4,5-C_10_Cl_4_ (4 consecutive, vicinal Cl). 1,1,1,3-C_10_Cl_4_ is the least polar of the measured CPs even though it contains one isolated –CHCl– group. This shows that CCl_3_ has a much smaller contribution to polarity than 3 × –CHCl–. It is interesting to note that ΔRI of 1,1,1,3-C_10_Cl_4_ is about half that of 1,1,1,3,9,10,10,10-C_10_Cl_8_. As the latter has double the CCl_3_–CH_2_–CHCl– substitution pattern, this observation suggests that the additivity principle may hold for the polarity of CPs, provided that the two structural units are far enough apart. The highest ΔRI of all congeners was observed for 1,2,5,6,9,10-C_10_Cl_6_ (3 pairs of vicinal Cl, of which 2 pairs at the ends). 1,5,5,6,6,10-C_10_Cl_6_ is the only CP standard with double chlorinated carbons and shows the second highest ΔRI. Comparison to 1,2,5,6,9,10-C_10_Cl_6_ suggests that –CCl_2_-CCl_2_- may be less polar than 4 chlorines all as vicinal –CHCl– groups. Overall, the total number of –CHCl– groups is not decisive for the polarity of CPs and the chlorination pattern needs to be considered.

### Describing polarity using ppLFERs

To investigate the types and the extent of polar interactions with CPs, ppLFER solute descriptors were derived. The *A* and *S* descriptors describe the polarity of the CPs relevant for GC retention times. First, the *E* values of CPs were obtained using the structure based estimation method from the UFZ-LSER database^25^, because *E* has been considered a simple additive property^16^. The *E* values obtained are presented in Supplementary Table S3. Second, *L* values were determined from SPB-Octyl data. The SPB-Octyl column exerts minimal polar interactions, and system parameters *s* and *a* were therefore set to 0. Thus,

3 $$ {\text{RI}} = c + eE + lL $$

Here, the measured RI values and the solute descriptors (*E*, *L*) of *n*-alcohols, *n*-alkylmethyl esters, *n*-alkanes and PAHs (Supplementary Table 3b) were used to calibrate system parameters (*c*, *e*, *l*) for SPB-Octyl by least-square multiple linear regression. The result is given in Supplementary Table S4. The solute descriptors for these chemicals were obtained from the UFZ-LSER database^25^. Then, from the system parameters and *E* and RI values of CPs, *L* values were calculated (Supplementary Table S3):

4 $$ L = \left( {{\text{RI}}{-}c{-}eE} \right)/l $$

The *A* and *S* solute descriptors of CPs were calculated from the rest of the data. The ppLFER model fit the calibration data well with *R*^2^ of 0.995–0.997 and the standard deviation (SD) of 36–59. System parameters for all columns were qualitatively in good agreement with those reported by Poole et al. using isothermal measurements (Supplementary Table S4). The *a* and *s* system parameters are in the order of the expected polarity of the columns: SPB-Octyl < HP-5ms <  < DB-17ms < InertCap-17ms <  < DB-225ms < SolGel-WAX. The ppLFER equations for the columns were transformed into:

5 $$ {\text{RI}}{-}c{-}eE{-}lL = sS + aA $$

*S* and *A* were determined from multiple linear regression with 0 intercept. The results are given in Supplementary Fig. S3. The standard errors of *S* and *A* were relatively high. This can be because of the incompatible results for the two most polar columns, DB-225ms and SolGel-WAX. As the *e*, *a* and *s* system parameters of the SolGel-WAX column are higher than those of the DB-225ms column, one would expect that RIs on the SolGel-WAX column would also be higher for all CPs. However, as Fig. 2 shows, RIs for SolGel-WAX were just as much as or even lower than those for DB-225ms. These conflicting results may cause a relatively large error in *A* and *S*.

**Figure 3:**
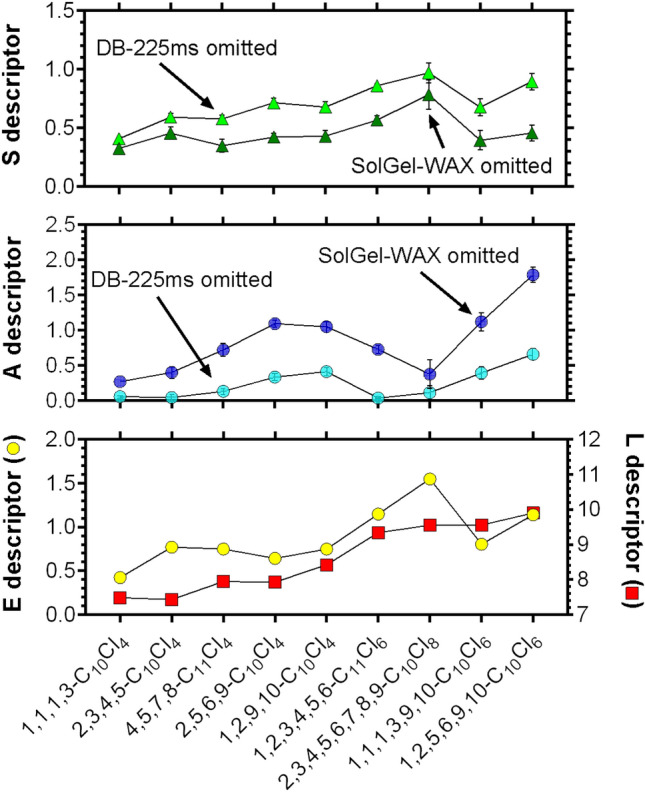
Solute descriptors *E*, *A*, *S* and *L* for a selection of CPs. *S* and *A* descriptors were determined using RI values from all columns while omitting either the SolGel-WAX or the DB-225ms column. Doing so has no influence on the determined *E* and *L* descriptor values.

As an attempt, we obtained *A* and *S* with the RI data for all but the SolGel-WAX column and for all but the DB-225ms column. While the resulting *A* and *S* descriptor values differ (on average 0.40 and 0.16, respectively), the trend across CP congeners remains the same between the two approaches (Fig. 3). The *S* descriptor generally increased with the number of chlorinated carbons. The lowest values were found for 1,1,1,3-C_10_Cl_4_ and highest for 1,2,5,6,9,10-C_10_Cl_6_ and 2,3,4,5,6,7,8,9-C_10_Cl_8_ (Fig. 3 and Supplementary Table S4). The *A* solute descriptor values were not related to the number of chlorines but rather to specific chlorination patterns. Substructures –CH_2_Cl and –CHCl– tend to increase *A*, but with the striking exception that compounds with consecutive –CHCl– structures (i.e., 2,3,4,5-C_10_Cl_4_ and 2,3,4,5,6,7,8,9-C_10_Cl_8_) had lower *A* descriptor values compared to CPs with the same number but a more distributed chlorination pattern (i.e., 4,5,7,8-C_11_Cl_4_, 2,5,6,9-C_10_Cl_4_ and 1,2,5,6,9,10-C_10_Cl_6_). The differences in ΔRI between constitutional isomers observed in the previous section are thus more related to H-bond donating properties (*A*) of the isomers.

The polar property of chlorinated carbon moieties stems from the high electronegativity of the Cl atom compared to that of the C atom. In a –CHCl– structure, the relatively high electron affinity of Cl has an inductive effect on C which results in a positive partial charge on the H atom. This makes the –CHCl– structure polar (positive *S*) and the H atom is then prone to act as a H-bond donor (positive *A*). Both properties allow for interactions with other polar compounds or phases like the polar GC columns. Such an inductive effect of Cl and the resulting H-bond donor property are well known for small chloroalkanes such as dichloromethane (*A* = 0.1) and chloroform (*A* = 0.15). However, in CP structures with vicinal –CHCl–, the Cl atom is often in proximity of the H atom of the neighboring –CHCl– structure which appear to diminish the ability of the H to fully act as a H-bond donor. Having 4 or more consecutive –CHCl– structures put each H atom in an even more crowded environment and brings back *A* to near 0 (Fig. 3). This interpretation is consistent with the existing knowledge on *A* for hexachlorocyclohexane (HCH) isomers. *A* values for *α*- and *γ*-HCHs are 0, whereas *β*-HCH poses a significant *A* value (0.12)^26^. Because of the different rotational configurations of the six –CHCl– units, *β*-HCH can take a conformation that maximizes the exposition of H atoms to the surrounding, whereas *α*- and *γ*-HCHs cannot do so.

A CCl_3_-CH_2_-CHCl- structure in 1,1,1,3-C_10_Cl_4_ has a minimal H-bonding property (see Fig. 3), which may be only attributable to the single –CHCl–. The –CCl_3_ group has no H-bond donor site and does not appear to make the neighboring –CH_2_– acidic (similar case for 1,1,1-trichloroethane with *A* = 0). However, a single Cl on the terminal carbon in a CH_2_Cl-CHCl- structure adds to H-bond donating properties of the CP (see *A* of 1,1,1,3-C_10_Cl_4_ < 1,1,1,3,9,10-C_10_Cl_6_). 1,2,3,4,5,6-Cl_10_Cl_6_ also contains this substructure although *A* is low, possibly due to steric effects or interference from the neighboring consecutive CHCl structure.

The inconsistent results for SolGel-WAX and DB-225ms can have several causes. For example, *n*-alkanes might undergo interfacial adsorption and can be retained under a mixed-mode retention mechanism on polar columns, which makes *n*-alkanes less suitable as reference compounds for determining RI values^27^. The exact reason is however difficult to conclude from the current data.

### COSMOthermX predictions

**Figure 4:**
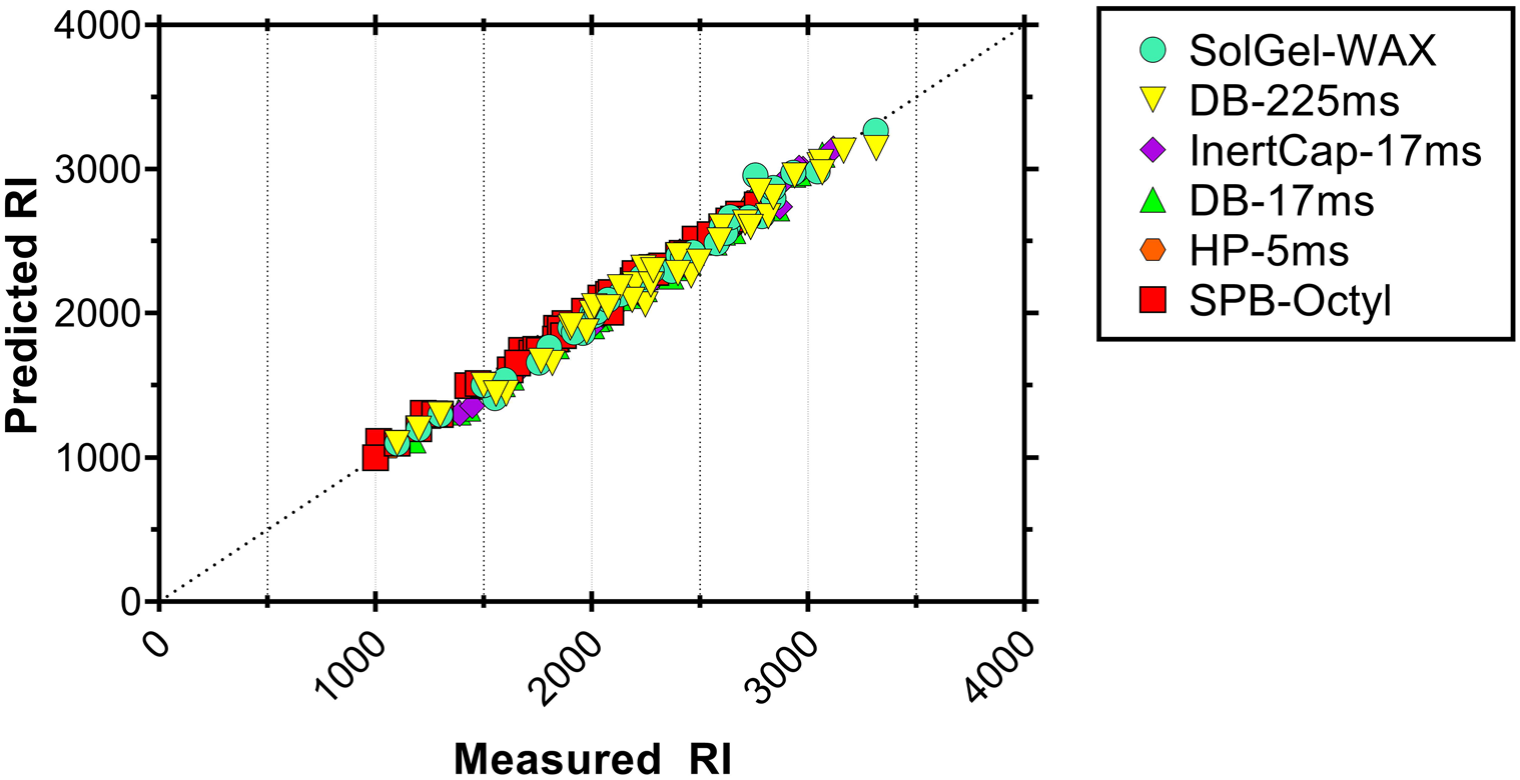
The RI values for CP congeners predicted by COSMO*thermX* for all columns in this study against the measured RI values from the GC system. Empirical corrections were applied to RI predictions (see text).

The COSMO*thermX*-predicted RIs correlated well with the measured RIs of CPs with an *R*^2^ between 0.975 and 0.995 (Supplementary Fig. S4). There is even high 1:1 agreement between predicted and measured RIs for SPB-Octyl, HP-5ms, and SolGel-WAX (RMSE: 44–72). The agreement, however, was lower for the columns DB-17ms, InertCap-17ms and DB-225ms (RMSE: 222–280). The CP group shows a trend that is not parallel to *n*-alkanes for these three columns (Supplementary Fig. S4), and thus the discrepancy increases with increasing RI value. The polymer coating of these columns contains a high proportion of phenyl groups (50% phenyl or diphenyl groups) and, apparently, the interaction properties of these groups with the CP structures is not fully captured by COSMO*thermX*. To make use of the high correlations between predicted and measured RIs, we applied an empirical correction to the predicted RI values by using the regression formula of predicted vs measured RI values for CPs (Supplementary Fig. S5). The results are shown in Fig. 4 and Supplementary Fig. S5. The RSME values after correction were between 21 and 75.

ΔRI values were calculated using the predicted RIs to test whether COSMO*therm*X can capture differences in polarity between CP congeners (Fig. 2C). Comparing Fig. 2B and 2C indicates that the overall trend agrees well with the experimentally observed ΔRIs. Thus, COSMO*thermX* correctly reflects polarity differences between CPs with differing chlorination patterns. The only discrepancy appears that COSMO*thermX* slightly overestimates the ΔRI values of CPs with many consecutive –CHCl– groups (i.e., 1,2,3,4,5,6-C_11_Cl_6_ and 2,3,4,5,6,7,8,9-C_10_Cl_8_). This statement however is conditional, because these two congeners have many possible diastereomers (16 and 70, respectively), for which COSMO*thermX* calculated a relatively wide range of ΔRIs. Currently, we do not know which diastereomers are present in the analytical standards.

### Effects of diastereomerism

**Figure 5:**
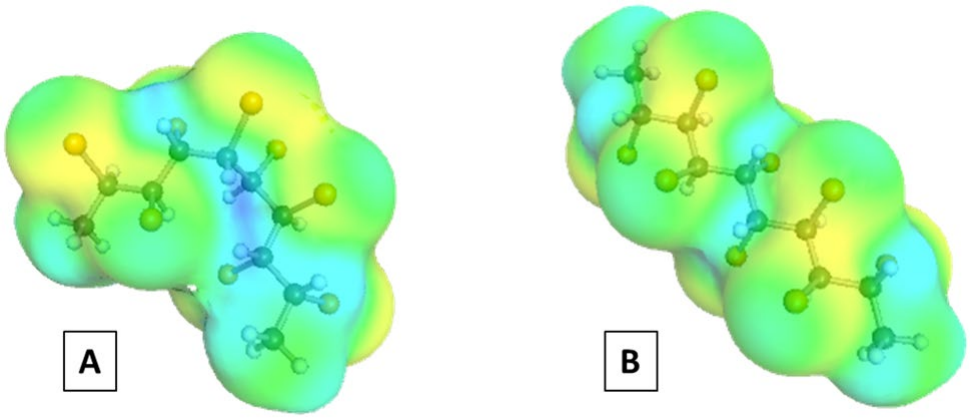
The lowest-energy conformers (_c0 suffix) of (2*R*,3*S*,4*S*,5*S*,6*S*,7*S*,8*S*,9*R*)-2,3,4,5,6,7,8,9-C_10_Cl_8_ (**A**) and (2*R*,3*R*,4*S*,5*S*,6*S*,7*S*,8*R*,9*R*)-2,3,4,5,6,7,8,9-C_10_Cl_8_ (**B**), generated by COSMO*confX*. Both are diastereomers of 2,3,4,5,6,7,8,9-C_10_Cl_8_ (this Figure was produced by the authors using the COSMO*view* software).

The range of predicted RI values by COSMO*thermX* shown in Supplementary Figs. S4 and S5 indicates the potential effects of diastereomerism of the CP on the partition properties (e.g., 2,3,4,5,6,7,8,9-C_10_Cl_8_). COSMO*thermX* predicts an increasing range of RI with increasing polarity of the polymer phase, which was also observed in the retention measurements on the GC systems. While CPs with many possible diastereomers usually showed a wide range in measured RI values, predicted RI values often span over an even wider range, suggesting that not all possible diastereomers are present in the CP standards. Comparing the two diastereomers of 2,3,4,5,6,7,8,9-C_10_Cl_8_, with the highest and lowest predicted RI values on the DB-225ms column ((2*R*,3*S*,4*S*,5*S*,6*S*,7*S*,8*S*,9*R*)-2,3,4,5,6,7,8,9-C_10_Cl_8_ and (2*R*,3*R*,4*S*,5*S*,6*S*,7*S*,8*R*,9*R*)-2,3,4,5,6,7,8,9-C_10_Cl_8_, predicted RI of 2721 and 2304, respectively), we can see that a difference in rotational configurations around the chiral carbons can result in distinctly different three-dimensional shapes (Fig. 5). Overall, according to the results from COSMO*thermX*, the difference between diastereomers can greatly affect the 3D-structure of the CP molecules, which, in turn, affects the interaction properties of the molecule and its partition behavior. Measurements for specific diastereomers would be interesting, although, unfortunately, such standards are currently unavailable and this study was able to present only a range of RI values for diastereomers.

## Conclusions

Inspection of RI values of CPs from GC columns with different polarity shows that the chlorination pattern plays an important role in determining polar interactions of CPs. Isolated –CHCl– groups or a pair of two vicinal –CHCl– are more polar than patterns with three or more consecutive –CHCl– groups. Polarity is also increased when a single Cl atom is present at the terminal carbon (e.g., –CH_2_Cl), whereas three Cl atoms at the terminal (–CCl_3_) add least to polarity of the CP molecules.

Determining ppLFER descriptors for CPs shows that polarity differs significantly between CP chlorination patterns and confirm the importance of Cl positioning to the H-bond donating properties (*A*) of CPs. The calculated solute descriptors show that H-bond interactions are lower for CPs with many consecutive –CHCl– groups than for CPs with a more distributed chlorination pattern.

Predictions from COSMO*thermX* show that the quantum chemically based modelling approach is capable of predicting RI values and can reflect the effect of variations in chlorination pattern on the interaction properties of CPs. This result supports the general accuracy of COSMO*thermX* to predict partition coefficients of CPs. While the prediction accuracy of the retention times is likely insufficient to allow the direct identification of specific CP congeners in real samples, retention time predictions by COSMO*thermX* for a diverse set of congeners could be compared to measured chromatograms of CPs to narrow down the possible structures present in the samples.

## Supplementary Information

Supplementary Information.

## Data Availability

The authors declare that all data supporting the findings of this study are available within the article and its supplementary information file.
