# Supplementary material for: Congener-specific partition properties of chlorinated paraffins evaluated with COSMOtherm and gas chromatographic retention indices
Source: Sci Rep. 2021 Feb 24;11:4426. doi: 10.1038/s41598-021-84040-z (PMC7904792; doi:10.1038/s41598-021-84040-z)
Supplement: Supplementary file 1 — Supplementary Information. [file 41598_2021_84040_MOESM1_ESM.docx]

**Congener-specific partition properties of chlorinated paraffins evaluated with COSMO*therm* and gas chromatographic retention indices**

Jort Hammer*, Hidenori Matsukami, Satoshi Endo

National Institute for Environmental Studies (NIES), Center for Health and Environmental Risk Research, Onogawa 16-2, 305-8506 Tsukuba, Ibaraki, Japan

*Corresponding author

**Supporting Information**

***Section S1: Prediction of RI using COSMOthermX***

The surrogate molecules of the column coatings used consisted of a monomer or an oligomer from the polymer structure (see Table 1 in the manuscript). For the quantum-mechanical calculations performed by COSMO*confX*, these surrogate molecules were end-capped with CH_3_ groups. Later, for the calculation of partition coefficients, these groups were disregarded using the weight string function in COSMO*thermX*. Since the combinatorial term in the free energy of partitioning is not well defined for polymers, all calculations were performed with the combinatorial term turned off. To predict RI values of all compounds, the calculated air-polymer partition coefficients (*K*_air/polymer_) from COSMO*thermX* need to be converted to RIs. While temperature-programmed RI is related but not directly proportional to *K*_air/polymer_,^1^ we opted a simple empirical approach for this conversion as described below.

As the GC measurements were performed under a program with linear temperature increase, the retention time corresponds to the elution temperature of each compound. In COSMO*thermX*, a linear relationship between log *K*_air/polymer_ and the reciprocal of temperature (1/*T*) was obtained for each compound by calculating partition coefficients at 5 different temperatures (Fig. S6). Using this linear relationship, the experimentally obtained elution temperatures for reference compounds (n-alkanes, n-alcohols, n-alkylmethylesters) were converted to log *K*_air/polymer_ values (Fig. S6A). The resulting log *K*_air/polymer_ values were specific to the column and were similar across the reference compounds (average SD = 0.15 in the current study). Note that compounds that eluted after the maximum temperature of the GC program was reached were removed from consideration. The mean of these “elution” log *K*_air/polymer_ values for reference compounds was calculated and was used to back-calculate the predicted elution temperatures for all compounds including CPs (Figure S6B). Finally, RI values were computed from these calculated elution temperatures using a modified version of equation 1 (i.e., where retention times were replaced by elution temperatures). Note that log *K*_air/polymer_ of long alkanes (> C_26_) were extrapolated from shorter alkanes because COMSO*confX* calculation times were too long (> 5 days).

***Section S2: GC-******APCI-QTOF-MS parameter optimization***

The APCI-QTOF-MS parameters were optimized for the identification of CP isomers, which were detected by APCI in positive mode. Intense pseudo-molecular ions of the CP isomers were observed corresponding to the dechlorinated and deprotonated molecules (Figs. S7a and S7b). Fragmentor voltage, capillary voltage, corona current and gas temperature for enhancing ionization (Fig. 8) were optimized and set to 150 V, 2500 V, 1.0 μA, and 350 °C, respectively.

**TABLES**

**Table S1.** The analytical standards of CP isomers used in this study. (N.A., not available)

| **Supplier** | **Cat No.** | **Compound name** | **Concentration**  **(μg/mL)** | **Purity**  **(%)** |
| --- | --- | --- | --- | --- |
| Dr Ehrenstorfer GmbH | DRE-LA17356500CY | 2,5,6,9-Tetrachlorodecane | 10 | 98.3 |
| Dr Ehrenstorfer GmbH | DRE-LA14171500CY | 1,2,5,6,9,10-Hexachlorodecane | 10 | 99.9 |
| Dr Ehrenstorfer GmbH | DRE-ZA15705000CY | 2,3,4,5,6,7,8,9-Octachlorodecane | 1 | 99.9 |
| Chiron AS | 1662.10-100-IO | 1,1,1,3-Tetrachlorodecane | 100 | 99.9 |
| Chiron AS | 1649.11-100-IO | 1,1,1,3-Tetrachloroundecane | 100 | 97.3 |
| Chiron AS | 1651.12-100-IO | 1,1,1,3-Tetrachlorododecane | 100 | 94.5 |
| Chiron AS | 1653.13-100-IO | 1,1,1,3-Tetrachlorotridecane | 100 | 97.1 |
| Chiron AS | 1676.14-K-IO | 1,1,1,3-Tetrachlorotetradecane | 1000 | N.A. |
| Chiron AS | 1622.10-100-IO | 1,1,1,3,8,10,10,10-Octachlorodecane | 100 | 96.4 |
| Chiron AS | 1623.11-100-IO | 1,1,1,3,9,11,11,11-Octachloroundecane | 100 | 99.9 |
| Chiron AS | 1624.12-100-IO | 1,1,1,3,10,12,12,12-Octachlorododecane | 100 | 97.7 |
| Chiron AS | 1625.13-100-IO | 1,1,1,3,11,13,13,13-Octachlorotridecane | 100 | 99.9 |
| Chiron AS | 1678.14-K-IO | 1,1,1,3,12,14,14,14-Octachlorotetradecane | 1000 | N.A. |
| Chiron AS | 1671.10-100-IO | 1,2,9,10-Tetrachlorodecane | 100 | 95.4 |
| Chiron AS | 1674.11-100-IO | 1,2,10,11-Tetrachloroundecane | 100 | N.A. |
| Chiron AS | 1677.14-K-IO | 1,2,13,14-Tetrachlorotetradecane | 1000 | N.A. |
| Chiron AS | 12285.11-100-IO | 1,2,3,4,5,6-Hexachloroundecane | 100 | 62.5 |
| Chiron AS | 12728.11-100-IO | 4,5,7,8- Tetrachloroundecane | 100 | 89.4 |
| Chiron AS | 12590.10-100-IO | 2,3,4,5-Tetrachlorodecane | 100 | 71.6 |
| Chiron AS | 12425.12-100-IO | 2,3,4,5-Tetrachlorododecane | 100 | 67.1 |
| CIL | CIL-ULM-8917-1.2 | 1,5,5,6,6,10-Hexachlorodecane | 100 | 95 |
| Chiron AS | 1659.10-100-IO | 1,1,1,3,9,10-Hexachlorodecane | 100 | 96.3 |
| Chiron AS | 1650.11-100-IO | 1,1,1,3,10,11-Hexachloroundecane | 100 | 99.9 |
| Chiron AS | 1652.12-100-IO | 1,1,1,3,11,12-Hexachlorododecane | 100 | 99.9 |
| Chiron AS | 1654.13-100-IO | 1,1,1,3,12,13-Hexachlorotridecane | 100 | 95 |

**Table S2**. Weighted mean and range of RI values of n-alcohols, n-alkylmethyl esters, PAHs and CPs for SPB-Octyl, HP-5ms, DB-17ms, InertCap-17ms, DB-225ms and SolGel-WAX colums. (N.A., not available)

| **Compounds**  **(number of possible diastereomers)** | **SPB-Octyl** | | **HP-5ms** | | **DB-17ms** | | **InertCap-17ms** | | **DB-225ms** | | **SolGel-WAX** | |
| --- | --- | --- | --- | --- | --- | --- | --- | --- | --- | --- | --- | --- |
|  | **Measured RI** | **Predicted RI** | **Measured RI** | **Predicted RI** | **Measured RI** | **Predicted RI** | **Measured RI** | **Predicted RI** | **Measured RI** | **Predicted RI** | **Measured RI** | **Predicted RI** |
| C_8_OH | 1015 | 1113 | 1068 | 1073 | 1174 | 1120 |  | 1106 | 1392 | 1205 | 1551 | 1413 |
| C_10_OH | 1220 | 1305 | 1272 | 1276 | 1382 | 1314 | 1388 | 1304 | 1604 | 1424 | 1755 | 1658 |
| C_12_OH | 1425 | 1496 | 1474 | 1474 | 1587 | 1508 | 1595 | 1507 | 1817 | 1633 | 1958 | 1863 |
| C_16_OH | 1836 | 1895 | 1882 | 1881 | 2000 | 1909 | 2009 | 1920 | 2247 | 2040 | 2370 | 2294 |
| C_18_OH | 2041 | 2104 | 2085 | 2087 | 2207 | 2119 | 2218 | 2136 | 2459 | 2245 | 2577 | 2486 |
| C_20_OH | 2245 | 2295 | 2289 | 2287 | 2410 | 2312 | 2434 | 2329 | 2673 | 2499 | 2786 | 2674 |
| C_8_OOC | 1071 | 1124 | 1123 | 1121 | 1226 | 1158 | 1229 | 1180 | 1347 | 1228 | 1393 | 1351 |
| C_10_OOC | 1273 | 1301 | 1324 | 1306 | 1427 | 1341 | 1446 | 1360 | 1556 | 1437 | 1596 | 1534 |
| C_12_OOC | 1474 | 1511 | 1525 | 1514 | 1628 | 1553 | 1644 | 1574 | 1765 | 1659 | 1802 | 1762 |
| C_14_OOC | 1675 | 1741 | 1726 | 1734 | 1836 | 1771 | 1847 | 1792 | 1975 | 1864 | 2008 | 1981 |
| C_16_OOC | 1876 | 1927 | 1927 | 1927 | 2040 | 1964 | 2050 | 1984 | 2186 | 2080 | 2215 | 2214 |
| C_18_OOC | 2076 | 2130 | 2128 | 2127 | 2243 | 2167 | 2254 | 2187 | 2397 | 2268 | 2421 | 2385 |
| C_20_OOC | 2277 | 2317 | 2330 | 2318 | 2447 | 2351 | 2458 | 2367 | 2607 | 2562 | 2630 | 2612 |
| C_22_OOC | 2477 | 2514 | 2531 | 2522 | 2652 | 2571 | 2662 | 2597 | 2816 | 2664 | 2838 | 2798 |
| C_24_OOC | 2678 | 2686 | 2733 | 2697 | 2856 | 2728 | 2868 | 2739 | 3023 | 2943 | 3043 | 2985 |
| Naphthalene | 1206 | 1198 | 1193 | 1156 | 1420 | 1420 | 1431 | 1214 | 1615 | 1288 | 1754 | 1469 |
| Acenaphthylene | 1489 | 1457 | 1463 | 1403 | 1767 | 1455 | 1795 | 1482 | 2048 | 1643 | 2209 | 1878 |
| Acenaphthene | 1529 | 1436 | 1498 | 1385 | 1796 | 1431 | 1827 | 1459 | 2027 | 1577 | 2156 | 1769 |
| Fluorene | 1630 | 1553 | 1598 | 1488 | 1914 | 1542 | 1951 | 1577 | 2196 | 1687 | 2353 | 1931 |
| Phenanthrene | 1854 | 1830 | 1800 | 1746 | 2192 | 1816 | 2247 | 1852 | 2571 | 2040 | 2747 | 2381 |
| Anthracene | 1865 | 1815 | 1810 | 1733 | 2206 | 1802 | 2257 | 1837 | 2578 | 2012 | 2752 | 2347 |
| Fluoranthene | 2163 | 2057 | 2087 | 1950 | 2569 | 2036 | 2644 | 2091 | 3010 | 2299 | 3221 | 2704 |
| Pyrene | 2236 | 2052 | 2143 | 1940 | 2667 | 2027 | 2749 | 2082 | 3090 | 2290 | 3319 | 2694 |
| Benz[a]anthracene | N.A. | 2316 | 2486 | 2255 | 3088 | 2337 | 3186 | 2391 | N.A. | 2596 | N.A. | 3000 |
| Chrysene | N.A. | 2426 | 2497 | 2304 | 3127 | 2396 | 3216 | 2458 | N.A. | 2713 | N.A. | 3253 |
| Benzo[*b*]fluoranthene | N.A. | 2543 | 2812 | 2392 | 3499 | 2488 | 3587 | 2564 | N.A. | 2718 | N.A. | 3294 |
| Benzo[*k*]fluoranthene | N.A. | 2528 | 2820 | 2387 | 3513 | 2480 | 3596 | 2552 | N.A. | 2750 | N.A. | 3267 |
| Benzo[*a*]pyrene | N.A. | 2550 | 2906 | 2392 | 3647 | 2490 | 3724 | 2569 | N.A. | 2721 | N.A. | 3302 |
| Dibenzo[*ah*]anthracene | N.A. | 2896 | 3224 | 2798 | N.A. | 2908 | 4072 | 2969 | N.A. | 3229 | N.A. | 3839 |
| Indeno[1,2,3-*cd*]pyrene | N.A. | 2729 | 3232 | 2675 | N.A. | 2770 | N.A. | 2831 | N.A. | 3074 | N.A. | 3572 |
| Benzo[*ghi*]perylene | N.A. | 2753 | 3290 | 2695 | N.A. | 2795 | 4179 | 2859 | N.A. | 3105 | N.A. | 3613 |

**Table S2**. (Continued)

| **Compounds**  **(number of diastereomers)** | **SPB-Octyl** | | **HP-5ms** | | **DB-17ms** | | **InertCap-17ms** | | **DB-225ms** | | **SolGel-WAX** | |
| --- | --- | --- | --- | --- | --- | --- | --- | --- | --- | --- | --- | --- |
|  | **Measured RI** | **Predicted RI** | **Measured RI** | **Predicted RI** | **Measured RI** | **Predicted RI** | **Measured RI** | **Predicted RI** | **Measured RI** | **Predicted RI** | **Measured RI** | **Predicted RI** |
| 1,1,1,3,9,10-C_10_Cl_6_ (4) | 2089  (2089 - 2089) | 2143  (2104 - 2161) | 2091  (2091 - 2091) | 2115  (2078 - 2132) | 2370  (2286 - 2396) | 2389  (2342 - 2410) | 2406  (2406 - 2406) | 2427  (2377 - 2450) | 2838  (2838 - 2838) | 2796  (2732 - 2826) | 2838  (2838 - 2839) | 2868  (2808 - 2895) |
| 1,1,1,3,10,11-C_11_Cl_6_ (4) | 2199  (2148 - 2204) | 2247  (2213 - 2272) | 2199  (2146 - 2202) | 2236  (2200 - 2264) | 2488  (2404 - 2488) | 2508  (2467 - 2541) | 2516  (2443 - 2522) | 2546  (2502 - 2582) | 2937  (2846 - 2950) | 2947  (2858 - 3011) | 2931  (2830 - 2949) | 2973  (2900 - 3034) |
| 1,1,1,3,11,12-C_12_Cl_6_ (4) | 2314  (2260 - 2315) | 2319  (2302 - 2358) | 2314  (2314 - 2314) | 2322  (2309 - 2357) | 2595  (2493 - 2631) | 2593  (2571 - 2641) | 2630  (2354 - 2636) | 2632  (2608 - 2685) | 3053  (2959 - 3057) | 3030  (2999 - 3089) | N.A. | 3031  (2981 - 3117) |
| 1,1,1,3,12,13-C_13_Cl_6_ (4) | 2418  (2369 - 2421) | 2415  (2400 - 2457) | 2407  (2163 - 2423) | 2420  (2407 - 2456) | 2709  (2562 - 2743) | 2706  (2687 - 2757) | 2743  (2628 - 2751) | 2751  (2730 - 2801) | 3163  (3067 - 3168) | 3126  (3103 - 3188) | N.A. | 3134  (3106 - 3207) |
| 1,1,1,3,14,15-C_15_Cl_6_ (4) | 2633  (2633 - 2633) | 2639  (2625 - 2664) | 2627  (2376 - 2663) | 2638  (2612 - 2683) | 2930  (2858 - 2969) | 2961  (2942 - 2993) | 2976  (2850 - 3020) | 3002  (2983 - 3036) | N.A. | 3352  (3317 - 3413) | N.A. | 3332  (3295 - 3399) |
| 1,1,1,3,8,10,10,10-C_10_Cl_8_ (3) | 2322  (2322 - 2322) | 2327  (2305 - 2340) | 2309  (2309 - 2324) | 2312  (2293 - 2324) | 2590  (2381 - 2627) | 2593  (2569 - 2607) | 2644  (2644 - 2644) | 2621  (2596 - 2637) | 3061  (3061 - 3061) | 3050  (3026 - 3064) | N.A. | 3097  (3066 - 3116) |
| 1,1,1,3,9,11,11,11-C_11_Cl_8_ (3) | 2437  (2437 - 2437) | 2403  (2393 - 2419) | 2425  (2409 - 2443) | 2393  (2384 - 2410) | 2715  (2499 - 2752) | 2683  (2672 - 2704) | 2758  (2585 - 2808) | 2719  (2707 - 2739) | N.A. | 3126  (3112 - 3144) | N.A. | 3177  (3159 - 3195) |
| 1,1,1,3,10,12,12,12-C_12_Cl_8_ (3) | 2546  (2546 - 2546) | 2545  (2512 - 2564) | 2526  (2272 - 2540) | 2523  (2502 - 2536) | 2763  (2763 - 2763) | 2858  (2828 - 2875) | 2883  (2693 - 2889) | 2911  (2878 - 2931) | N.A. | 3285  (3262 - 3297) | N.A. | 3319  (3298 - 3331) |
| 1,1,1,3,11,13,13,13-C_13_Cl_8_ (3) | 2654  (2654 - 2654) | 2652  (2648 - 2655) | 2620  (2381 - 2651) | 2639  (2633 - 2646) | 2955  (2927 - 3009) | 2971  (2967 - 2976) | 2958  (2686 - 3046) | 3009  (3004 - 3013) | N.A. | 3396  (3388 - 3402) | N.A. | 3414  (3408 - 3420) |
| 1,1,1,3,12,14,14,14-C_14_Cl_8_ (3) | 2764  (2764 - 2764) | 2751  (2718 - 2793) | 2746  (2490 - 2762) | 2774  (2747 - 2807) | 3065  (2780 - 3108) | 3100  (3060 - 3148) | 3116  (2994 - 3160) | 3142  (3101 - 3192) | N.A. | 3554  (3507 - 3611) | N.A. | 3552  (3509 - 3607) |
| 1,1,1,3-C_10_Cl_4_ (2) | 1622  (1606 - 1856) | 1606  (1606 - 1606) | 1615  (1615 - 1615) | 1615  (1615 - 1615) | 1734  (1734 - 1849) | 1748  (1748 - 1748) | 1749  (1749 - 1749) | 1760  (1760 - 1760) | 1906  (1828 - 2094) | 1918  (1918 - 1918) | 1916  (1908 - 1917) | 1866  (1866 - 1866) |
| 1,1,1,3-C_11_Cl_4_ (2) | 1721  (1651 - 1723) | 1723  (1723 - 1723) | 1721  (1721 - 1721) | 1734  (1734 - 1734) | 1844  (1763 - 1844) | 1885  (1885 - 1885) | 1856  (1856 - 1856) | 1899  (1899 - 1899) | 2011  (1930 - 2012) | 2054  (2054 - 2054) | 2022  (1994 - 2024) | 2010  (2010 - 2010) |
| 1,1,1,3-C_12_Cl_4_ (2) | 1832  (1800 - 1896) | 1819  (1819 - 1820) | 1826  (1826 - 1826) | 1843  (1842 - 1844) | 1948  (1755 - 2063) | 1999  (1998 - 2000) | 1965  (1939 - 2036) | 2012  (2010 - 2013) | 2130  (2053 - 2315) | 2183  (2180 - 2186) | 2132  (2132 - 2132) | 2132  (2128 - 2136) |
| 1,1,1,3-C_13_Cl_4_ (2) | 1934  (1913 - 1935) | 1908  (1908 - 1908) | 1932  (1932 - 1932) | 1934  (1934 - 1934) | 2036  (1955 - 2036) | 2106  (2106 - 2106) | 2072  (2072 - 2072) | 2122  (2122 - 2122) | 2238  (2220 - 2239) | 2319  (2319 - 2319) | 2237  (2208 - 2240) | 2232  (2232 - 2232) |
| 1,1,1,3-C_14_Cl_4_ (2) | 2042  (2042 - 2042) | 2029  (2025 - 2032) | 2038  (1941 - 2111) | 2051  (2048 - 2053) | 2162  (1966 - 2264) | 2245  (2242 - 2248) | 2183  (1979 - 2291) | 2266  (2263 - 2269) | N.A. | 2468  (2464 - 2472) | N.A. | 2333  (2330 - 2337) |
| 1,2,9,10-C_10_Cl_4_ 2) | 1853  (1802 - 1855) | 1890  (1887 - 1893) | 1876  (1876 - 1876) | 1894  (1893 - 1896) | 2132  (2065 - 2132) | 2105  (2101 - 2109) | 2167  (2167 - 2167) | 2141  (2138 - 2145) | 2590  (2314 - 2601) | 2500  (2492 - 2509) | 2617  (2507 - 2621) | 2558  (2542 - 2574) |

**Table S2**. (Continued)

| **Compounds** | **SPB-octyl** |  | **HP5** |  | **DB17** |  | **Inert17** |  | **DB225** |  | **WAX** |  |
| --- | --- | --- | --- | --- | --- | --- | --- | --- | --- | --- | --- | --- |
|  | **Measured RI** | **Predicted RI** | **Measured RI** | **Predicted RI** | **Measured RI** | **Predicted RI** | **Measured RI** | **Predicted RI** | **Measured RI** | **Predicted RI** | **Measured RI** | **Predicted RI** |
| 1,2,10,11-C_11_Cl_4_ (2) | 1965  (1918 - 1987) | 2014  (2007 - 2020) | 1985  (1931 - 2158) | 2008  (2002 - 2014) | 2247  (2177 - 2283) | 2248  (2240 - 2257) | 2277  (2277 - 2277) | 2291  (2282 - 2299) | 2708  (2615 - 2787) | 2624  (2614 - 2634) | 2724  (2620 - 2785) | 2664  (2653 - 2675) |
| 1,2,13,14-C_14_Cl_4_(2) | 2296  (2296 - 2296) | 2282  (2278 - 2286) | 2312  (2309 - 2485) | 2304  (2300 - 2308) | 2578  (2578 - 2809) | 2551  (2546 - 2557) | 2645  (2388 - 2857) | 2590  (2583 - 2597) | N.A. | 2921  (2907 - 2935) | N.A. | 2883  (2874 - 2891) |
| 2,5,6,9-C_10_Cl_4_ (6) | 1740  (1734 - 1745) | 1748  (1639 - 1820) | 1782  (1769 - 1787) | 1754  (1645 - 1825) | 2022  (2014 - 2031) | 1958  (1839 - 2035) | 2053  (2043 - 2059) | 1992  (1874 - 2068) | 2495  (2437 - 2511) | 2343  (2196 - 2456) | 2467  (2421 - 2482) | 2421  (2306 - 2537) |
| 4,5,7,8-C_11_Cl_4_ (6) | 1757  (1738 - 1761) | 1741  (1678 - 1778) | 1774  (1749 - 1782) | 1751  (1698 - 1787) | 1934  (1908 - 1954) | 1931  (1869 - 1969) | 1970  (1962 - 1981) | 1955  (1895 - 1992) | 2272  (2212 - 2290) | 2197  (2117 - 2251) | 2231  (2165 - 2256) | 2250  (2187 - 2291) |
| 1,2,5,6,9,10-C_10_Cl_6_ (6) | 2199  (2199 - 2200) | 2274  (2229 - 2300) | 2231  (2229 - 2231) | 2243  (2196 - 2282) | 2605  (2598 - 2605) | 2560  (2501 - 2594) | 2659  (2659 - 2659) | 2597  (2532 - 2640) | 3314  (3314 - 3314) | 3140  (3053 - 3185) | 3312  (3311 - 3313) | 3267  (3167 - 3322) |
| 2,3,4,5,6,7,8,9-C_10_Cl_8_ (72) | 2181  (2177 - 2199) | 2153  (2031 - 2307) | 2201  (2199 - 2213) | 2171  (2041 - 2335) | 2461  (2461 - 2461) | 2419  (2263 - 2610) | 2525  (2525 - 2525) | 2480  (2293 - 2692) | 2773  (2769 - 2817) | 2851  (2587 - 3127) | 2756  (2754 - 2770) | 2956  (2622 - 3300) |
| 1,2,3,4,5,6-C_11_Cl_6_ (16) | 2088  (2030 - 2114) | 2006  (1936 - 2109) | 2101  (1918 - 2253) | 2040  (1961 - 2104) | 2366  (2245 - 2391) | 2254  (2150 - 2348) | 2391  (2339 - 2431) | 2308  (2181 - 2389) | 2734  (2587 - 2842) | 2609  (2454 - 2695) | 2639  (2500 - 2733) | 2665  (2447 - 2780) |
| 2,3,4,5-C_10_Cl_4_ (6) | 1656  (1612 - 1677) | 1654  (1539 - 1704) | 1667  (1619 - 1692) | 1662  (1557 - 1699) | 1841  (1750 - 1856) | 1820  (1680 - 1878) | 1844  (1773 - 1884) | 1841  (1694 - 1893) | 2075  (1915 - 2162) | 2042  (1847 - 2119) | 2074  (1925 - 2148) | 2092  (1819 - 2203) |
| 2,3,4,5-C_12_Cl_4_ (6) | 1868  (1827 - 1890) | 1842  (1757 - 1891) | 1875  (1831 - 1900) | 1868  (1783 - 1905) | 2060  (1962 - 2060) | 2041  (1930 - 2103) | 2055  (1987 - 2095) | 2062  (1946 - 2121) | 2283  (2142 - 2387) | 2298  (2089 - 2401) | 2276  (2141 - 2355) | 2292  (2054 - 2418) |
| 1,5,5,6,6,10-C_10_Cl_6_ (1) | 2190  (2190 - 2190) | 2226  (2226 - 2226) | 2214  (2214 - 2214) | 2209  (2209 - 2209) | 2566  (2566 - 2566) | 2489  (2489 - 2489) | 2619  (2619 - 2619) | 2544  (2544 - 2544) | 3065  (3065 - 3065) | 2965  (2965 - 2965) | N.A. | 2998  (2998 - 2998) |

**Table S3a.** The ppLFER solute descriptors for CPs whereof data was available from all columns (with an exception for 1,5,5,6,6,10-C_10_Cl_6_, which was not detected on the SolGel-WAX). E values for CPs were obtained from the UFZ database and L was calculated using the SPB-Octyl column. A and S values were calculated using either all columns without the DB-225ms column or without the SolGel-WAX column (see text for more details). E, A, S and L values for n-alcohols, n-alkylmethyl esters, n-alkanes and PAHs were obtained from the UFZ database. (N.A., not available)

| Compounds | E | A  (without SolGel-WAX) | A  (without DB-225ms) | S  (without SolGel-WAX) | S  (without DB-225ms) | L |
| --- | --- | --- | --- | --- | --- | --- |
| 1,1,1,3,10,11-C_11_Cl_6_ | 0.85 | 1.062 (±0.148) | 0.353 (±0.091) | 0.417 (±0.092) | 0.692 (±0.080) | 10.104 |
| 1,1,1,3,11,12-C_12_Cl_6_ | 0.85 | 1.092 (±0.139) | -1.10 (±0.119) | 0.392 (±0.086) | 1.241 (±0.104) | 10.674 |
| 1,1,1,3,12,13-C_13_Cl_6_ | 0.85 | 1.086 (±0.198) | -1.11 (±0.150) | 0.404 (±0.124) | 1.256 (±0.131) | 11.190 |
| 1,1,1,3,8,10,10,10-C_10_Cl_8_ | 1.04 | 1.124 (±0.165) | -1.08 (±0.136) | 0.370 (±0.103) | 1.227 (±0.119) | 10.679 |
| 1,1,1,3,9,10-C_10_Cl_6_ | 0.85 | 1.119 (±0.130) | 0.391 (±0.083) | 0.393 (±0.081) | 0.676 (±0.073) | 9.558 |
| 1,1,1,3-C_10_Cl_4_ | 0.52 | 0.267 (±0.026) | 0.055 (±0.018) | 0.323 (±0.016) | 0.406 (±0.016) | 7.483 |
| 1,1,1,3-C_11_Cl_4_ | 0.52 | 0.231 (±0.019) | 0.040 (±0.014) | 0.357 (±0.011) | 0.432 (±0.012) | 7.975 |
| 1,1,1,3-C_12_Cl_4_ | 0.52 | 0.297 (±0.032) | 0.060 (±0.022) | 0.315 (±0.020) | 0.407 (±0.019) | 8.524 |
| 1,1,1,3-C_13_Cl_4_ | 0.52 | 0.337 (±0.070) | 0.079 (±0.039) | 0.290 (±0.043) | 0.390 (±0.034) | 9.030 |
| 1,2,10,11-C_11_Cl_4_ | 0.65 | 1.079 (±0.086) | 0.405 (±0.059) | 0.413 (±0.054) | 0.675 (±0.051) | 8.978 |
| 1,2,3,4,5,6-C_11_Cl_6_ | 1.14 | 0.725 (±0.062) | -0.03 (±0.015) | 0.565 (±0.039) | 0.858 (±0.013) | 9.338 |
| 1,2,5,6,9,10-C_10_Cl_6_ | 0.99 | 1.787 (±0.107) | 0.656 (±0.080) | 0.455 (±0.067) | 0.893 (±0.069) | 9.898 |
| 1,2,9,10-C_10_Cl_4_ | 0.65 | 1.049 (±0.077) | 0.411 (±0.054) | 0.429 (±0.048) | 0.677 (±0.047) | 8.424 |
| 1,5,5,6,6,10-C_10_Cl_6_ | 0.81 | 1.045 (±0.000) | N.A. | 0.647 (±0.000) | N.A. | 10.054 |
| 2,3,4,5,6,7,8,9-C_10_Cl_8_ | 1.56 | 0.373 (±0.202) | -0.11 (±0.098) | 0.782 (±0.126) | 0.968 (±0.085) | 9.551 |
| 2,3,4,5-C_10_Cl_4_ | 0.74 | 0.398 (±0.084) | 0.042 (±0.036) | 0.454 (±0.052) | 0.590 (±0.031) | 7.433 |
| 2,3,4,5-C_12_Cl_4_ | 0.74 | 0.381 (±0.085) | 0.028 (±0.038) | 0.454 (±0.053) | 0.590 (±0.033) | 8.490 |
| 2,5,6,9-C_10_Cl_4_ | 0.59 | 1.092 (±0.051) | 0.333 (±0.715) | 0.420 (±0.031) | 0.714 (±0.037) | 7.930 |
| 4,5,7,8-C_11_Cl_4_ | 0.65 | 0.720 (±0.088) | 0.129 (±0.038) | 0.347 (±0.055) | 0.575 (±0.033) | 7.946 |

**Table S3b.** The solute descriptors E, A, S and L for the reference compounds used for the determination of system parameters for the GC-columns in this study.

| Compound | E | A | S | L | Compound | E | A | S | L |
| --- | --- | --- | --- | --- | --- | --- | --- | --- | --- |
| C_8_OH | 0.2 | 0.37 | 0.42 | 4.619 | C_6_OOC | 0.08 | 0 | 0.6 | 3.874 |
| C_10_OH | 0.19 | 0.37 | 0.42 | 5.628 | C_8_OOC | 0.07 | 0 | 0.6 | 4.838 |
| C_12_OH | 0.18 | 0.37 | 0.42 | 6.62 | C_10_OOC | 0.05 | 0 | 0.6 | 5.803 |
| C_16_OH | 0.15 | 0.37 | 0.42 | 8.654 | C_12_OOC | 0.04 | 0 | 0.6 | 6.767 |
| C_18_OH | 0.15 | 0.37 | 0.42 | 9.662 | C_14_OOC | 0.03 | 0 | 0.6 | 7.731 |
| C_20_OH | 0.14 | 0.37 | 0.42 | 10.667 | C_16_OOC | 0.02 | 0 | 0.6 | 8.695 |
| Decane | 0 | 0 | 0 | 4.686 | C_18_OOC | 0.01 | 0 | 0.6 | 9.659 |
| Undecane | 0 | 0 | 0 | 5.191 | C_20_OOC | 0 | 0 | 0.6 | 10.75 |
| Dodecane | 0 | 0 | 0 | 5.696 | C_22_OOC | 0.05 | 0 | 0.6 | 11.82 |
| Tridecane | 0 | 0 | 0 | 6.2 | C_24_OOC | 0.05 | 0 | 0.6 | 12.824 |
| Tetradecane | 0 | 0 | 0 | 6.705 | Naphthalene | 1.23 | 0 | 0.91 | 5.157 |
| Pentadecane | 0 | 0 | 0 | 7.209 | Acenaphthylene | 1.55 | 0 | 1.13 | 6.395 |
| Hexadecane | 0 | 0 | 0 | 7.714 | Acenaphthene | 1.45 | 0 | 0.95 | 6.709 |
| Heptadecane | 0 | 0 | 0 | 8.218 | Fluorene | 1.66 | 0 | 1.1 | 6.948 |
| Octadecane | 0 | 0 | 0 | 8.722 | Phenanthrene | 1.92 | 0 | 1.28 | 7.712 |
| Nonadecane | 0 | 0 | 0 | 9.226 | Anthracene | 1.98 | 0 | 1.28 | 7.735 |
| Eicosane | 0 | 0 | 0 | 9.731 | Fluoranthene | 2.35 | 0 | 1.48 | 8.733 |
| Henicosane | 0 | 0 | 0 | 10.236 | Pyrene | 2.24 | 0 | 1.48 | 8.974 |
| Docosane | 0 | 0 | 0 | 10.74 | Benz[*a*]anthracene | 2.74 | 0 | 1.68 | 10.124 |
| Tricosane | 0 | 0 | 0 | 11.252 | Chrysene | 2.65 | 0 | 1.67 | 10.123 |
| Tetracosane | 0 | 0 | 0 | 11.758 | Benzo[*b*]fluoranthene | 3.19 | 0 | 1.82 | 11.632 |
| Pentacosane | 0 | 0 | 0 | 12.264 | Benzo[*k*]fluoranthene | 3.19 | 0 | 1.91 | 11.607 |
| Hexacosane | 0 | 0 | 0 | 12.77 | Benzo[*a*]pyrene | 3.02 | 0 | 1.85 | 11.54 |
| Heptacosane | 0 | 0 | 0 | 13.276 |  |  |  |  |  |
| Octacosane | 0 | 0 | 0 | 13.78 |  |  |  |  |  |
| Nonacosane | 0 | 0 | 0 | 14.291 |  |  |  |  |  |
| Triacontane | 0 | 0 | 0 | 14.794 |  |  |  |  |  |

**Table S4**. System parameters e, a, s, l and c, and SD and R^2^ for the columns in this study calculated using reference compounds. Values in parentheses are standard errors.

|  | System parameters | | | | |  |  |
| --- | --- | --- | --- | --- | --- | --- | --- |
| Compounds | **e** | **a** | **s** | **l** | **c** | **SD** | **R^2^** |
| SPB-Octyl | 126 (±8) | 0 | 0 | 202 (±1) | 60 (±21) | 36 | 0.997 |
| HP-5ms | 8 (±16) | 212 (±27) | 73 (±48) | 203 (±1) | 7 (±21) | 39 | 0.996 |
| DB-17ms | 113 (±23) | 401 (±40) | 155 (±72) | 205 (±2) | -15 (±29) | 59 | 0.994 |
| InertCap-17ms | 123 (±26) | 438 (±46) | 175 (±84) | 205 (±2) | -33 (±35) | 62 | 0.993 |
| DB-225ms | 81 (±20) | 644 (±31) | 507 (±55) | 206 (±2) | -11 (±27) | 44 | 0.994 |
| SolGel-WAX | 143 (±18) | 691 (±29) | 779 (±52) | 203 (±2) | 14 (±24) | 41 | 0.995 |

**FIGURES**

**
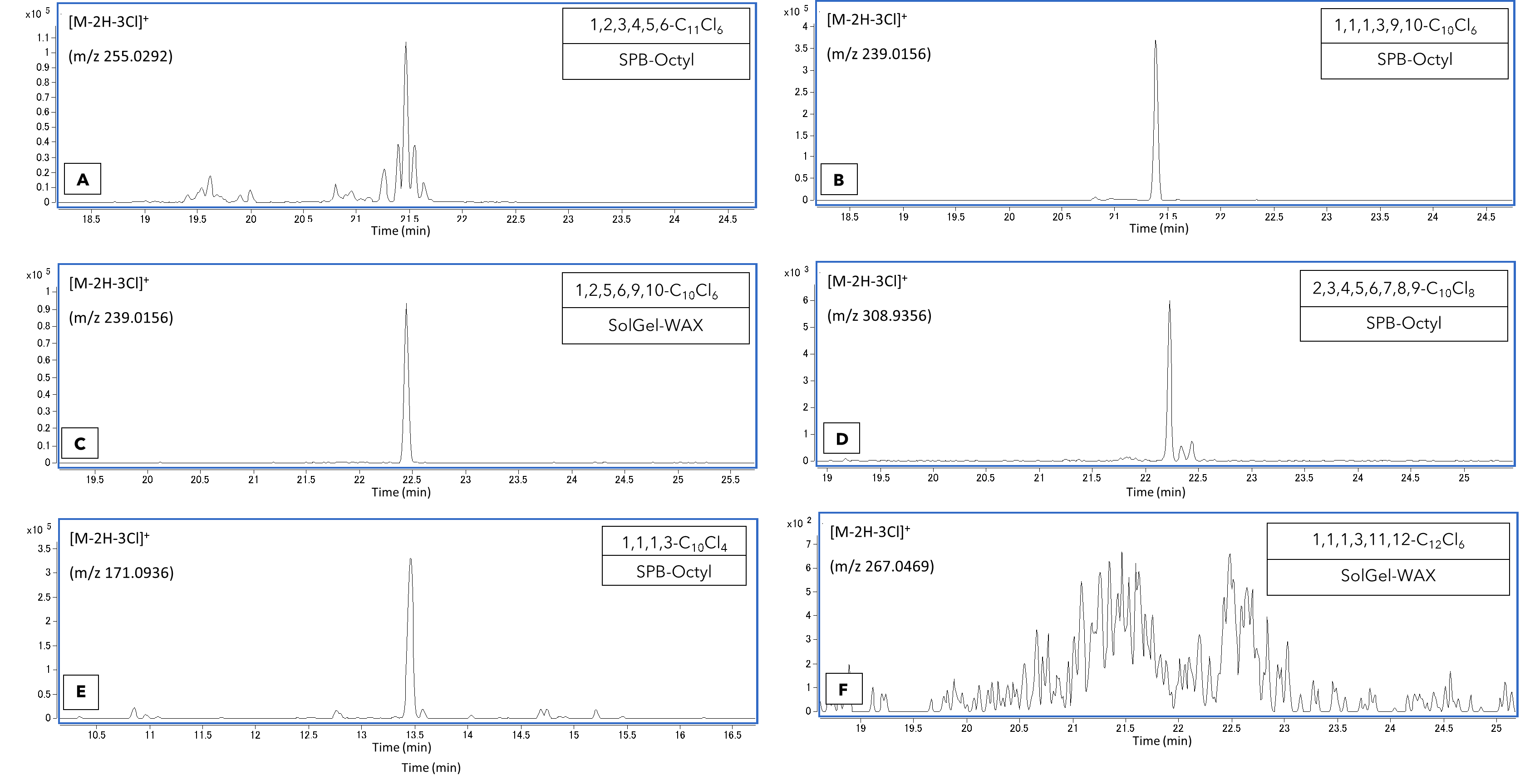
**

**Figure S1**. Chromatograms of several CP standards on the SPB-Octyl column (A,B,D,E) and the SolGel-WAX column (C,F).

**
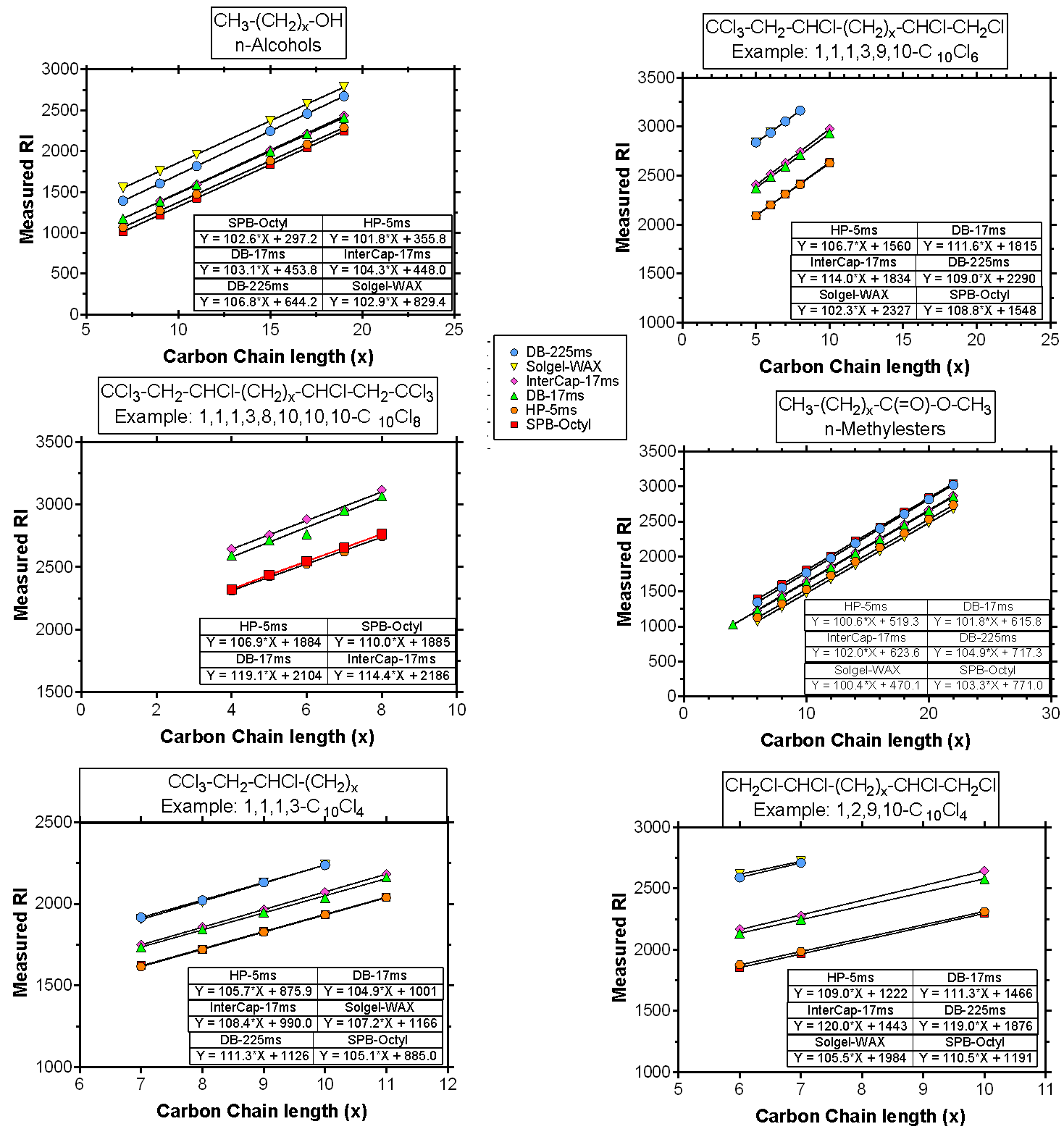
 Figure S2**. RI values against carbon chain length for n-alcohols, n-alkylmethyl esters and 4 groups of CPs with different chlorination patterns.

**Figure S3**. Solute descriptors for a selection of CPs calculated using measured RI values from all columns in this study (including both DB-225ms and SolGel-WAX). Error bars indicate the standard errors from multiple linear regression analysis.


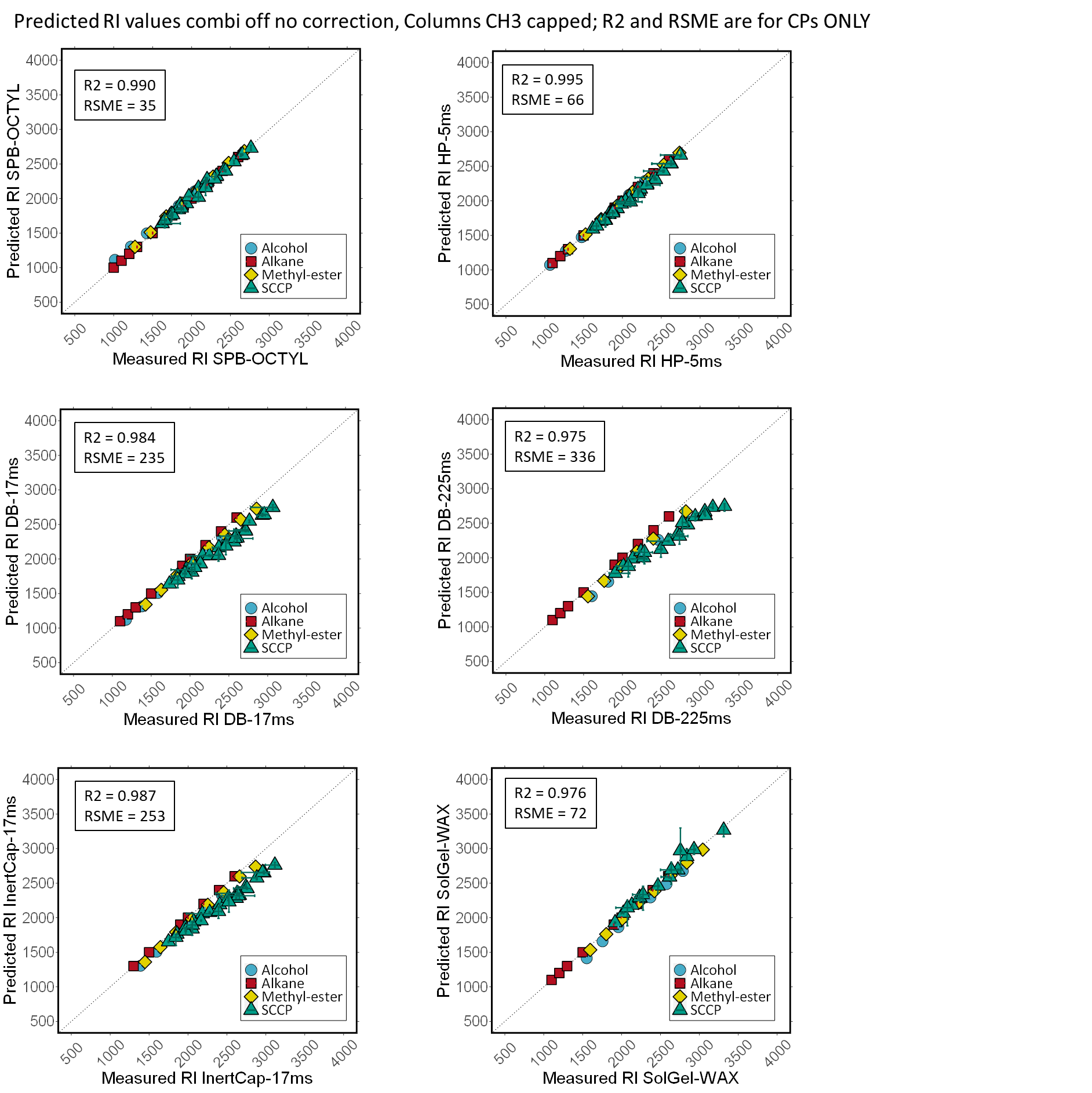


**Figure S4**. Predicted RI values from COSMO-RS vs measured RI values for all compounds. The vertical and horizontal error bars show the range of measured RIs for multiple peaks.


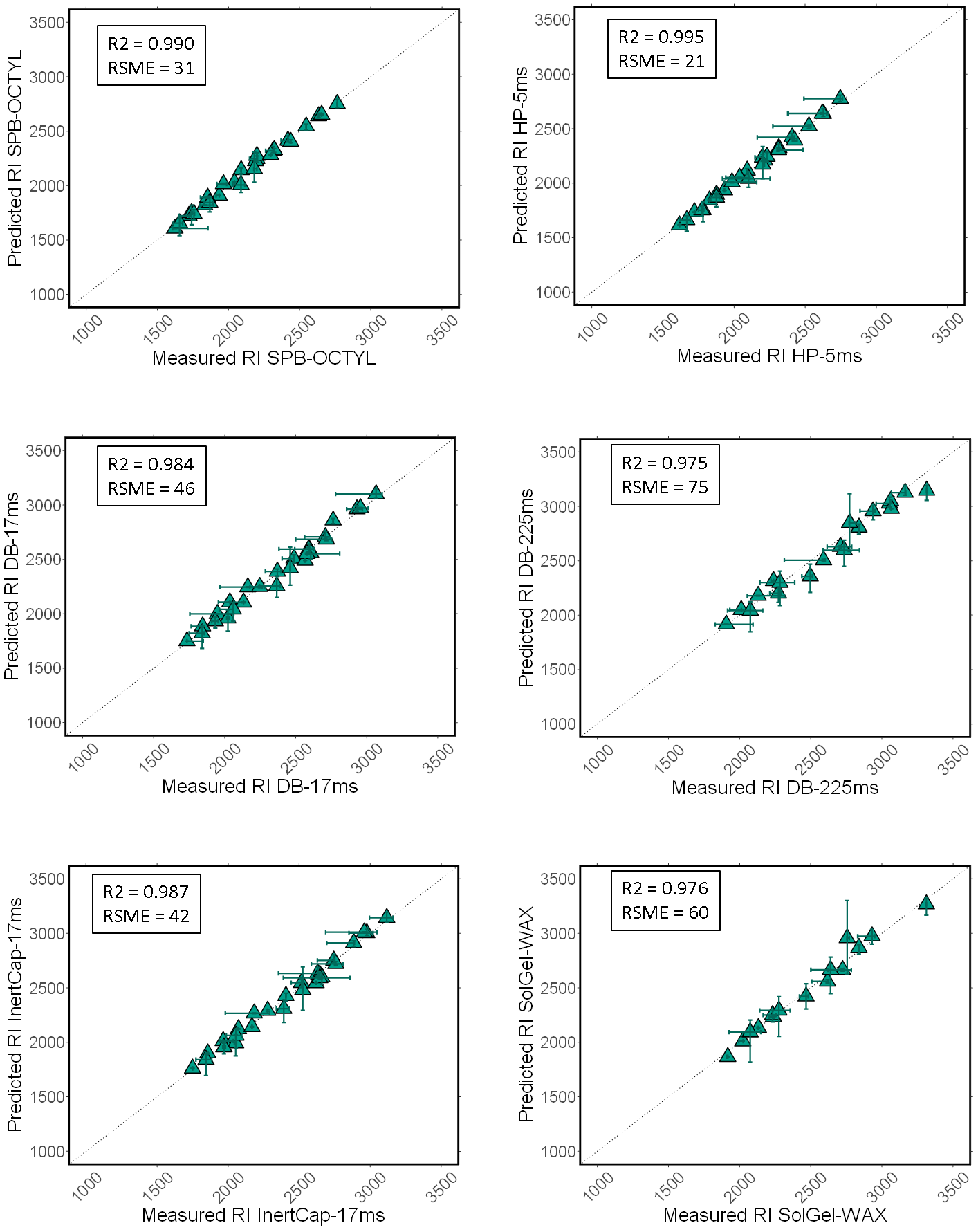


**Figure S5**. Predicted RI values with corrections vs measured RI values for CPs. The vertical and horizontal error bars show the range of measured RIs for multiple peaks.


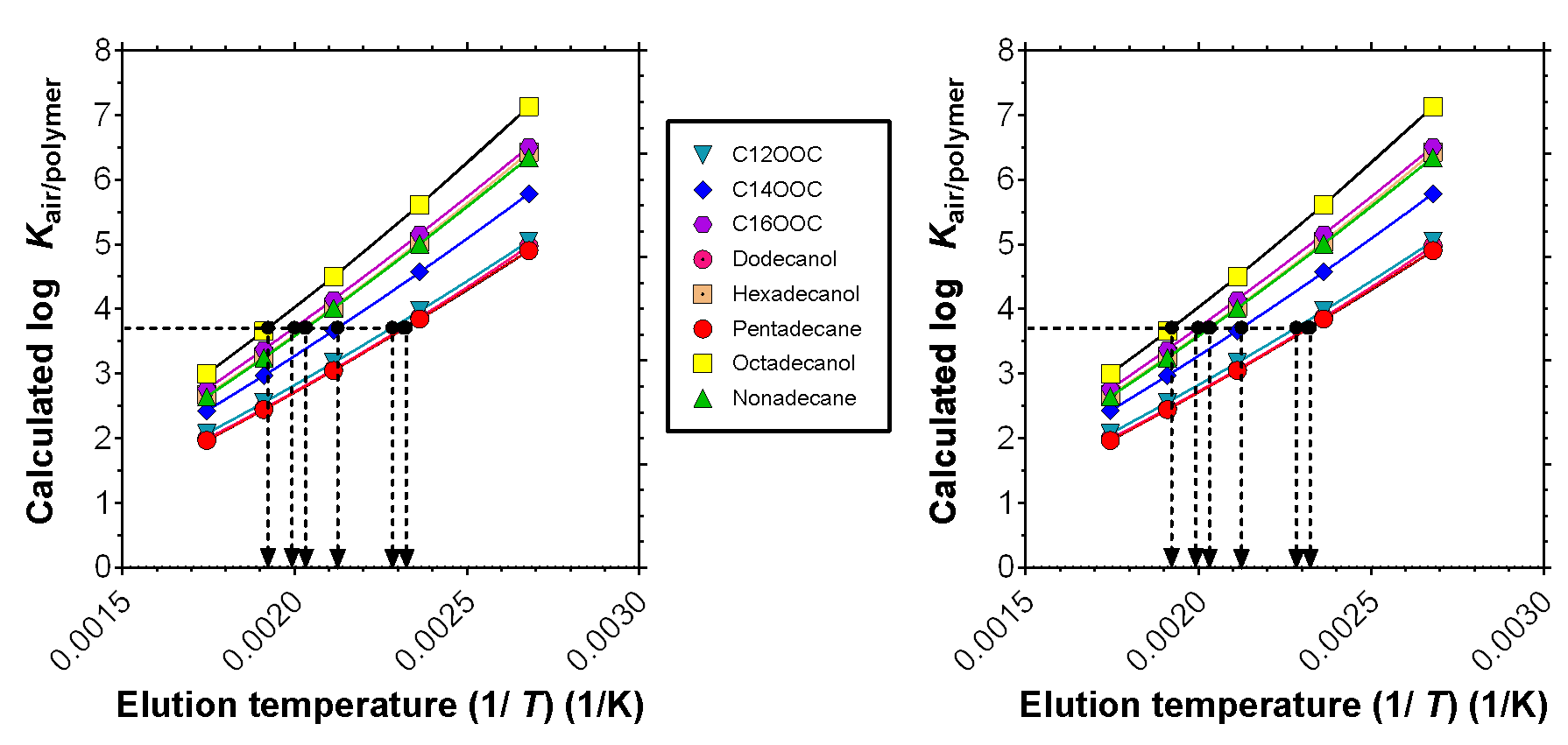


**Figure S6**. Calculated log *K*_air/polymer_ partition coefficients from COSMO*thermX* versus the elution temperature. Panel A shows how the elution temperatures obtained from the retention time and the applied GC temperature program were used to derive the partition coefficient for the reference compounds. Panel B shows how the average *K*_air/polymer_ partition coefficient is used to back-calculate the elution temperature for each compound.

**Figure S7a.** Mass spectra of (A) 1,1,1,3-C_10_Cl_4_, (B) 2,5,6,9-C_10_Cl_4_, (C) 1,2,9,10-C_10_Cl_4_, and (D) 1,1,1,3,9,10-C_10_Cl_6_ obtained by GC-APCI-TOF-MS analysis.

**Figure S7b**. Mass spectra of (E) 1,2,5,6,9,10-C_10_Cl_6_, (F) 1,5,5,6,6,10-C_10_Cl_6_, (G) 1,1,1,3,8,10,10,10-C_10_Cl_8_, and (H) 2,3,4,5,6,7,8,9-C_10_Cl_8_ obtained by GC-APCI-TOF-MS analysis.

**Figure S8.** Relationship between abundances of CP isomer peaks and values of (A) fragmentor voltage, (B) capillary voltage, (C) corona current, and (D) gas temperature of APCI-TOF-MS system.

Reference

1. Curvers, J., Rijks, J., Cramers, C., Knauss, K. & Larson, P. Temperature programmed retention indices: Calculation from isothermal data. Part 1: Theory. *J. High Resolut. Chromatogr.* **8**, 607–610 (1985).
